# Cleavage of the SUN-domain protein Mps3 at its N-terminus regulates centrosome disjunction in budding yeast meiosis

**DOI:** 10.1371/journal.pgen.1006830

**Published:** 2017-06-13

**Authors:** Ping Li, Hui Jin, Bailey A. Koch, Rebecca L. Abblett, Xuemei Han, John R. Yates, Hong-Guo Yu

**Affiliations:** 1 Department of Biological Science, the Florida State University, Tallahassee, FL, United States of America; 2 Zhejiang Gongshang University, Key Laboratory for Food Microbial Technology, Hangzhou, China; 3 The Scripps Research Institute, LaJolla, CA, United States of America; The University of North Carolina at Chapel Hill, UNITED STATES

## Abstract

Centrosomes organize microtubules and are essential for spindle formation and chromosome segregation during cell division. Duplicated centrosomes are physically linked, but how this linkage is dissolved remains unclear. Yeast centrosomes are tethered by a nuclear-envelope-attached structure called the half-bridge, whose components have mammalian homologues. We report here that cleavage of the half-bridge protein Mps3 promotes accurate centrosome disjunction in budding yeast. Mps3 is a single-pass SUN-domain protein anchored at the inner nuclear membrane and concentrated at the nuclear side of the half-bridge. Using the unique feature in yeast meiosis that centrosomes are linked for hours before their separation, we have revealed that Mps3 is cleaved at its nucleus-localized N-terminal domain, the process of which is regulated by its phosphorylation at serine 70. Cleavage of Mps3 takes place at the yeast centrosome and requires proteasome activity. We show that noncleavable Mps3 (Mps3-nc) inhibits centrosome separation during yeast meiosis. In addition, overexpression of *mps3-nc* in vegetative yeast cells also inhibits centrosome separation and is lethal. Our findings provide a genetic mechanism for the regulation of SUN-domain protein-mediated activities, including centrosome separation, by irreversible protein cleavage at the nuclear periphery.

## Author summary

The nucleus, where the eukaryotic chromosomes are stored, is enclosed by a double-membrane structure called the nuclear envelope. Located at the inner nuclear membrane, a class of highly conserved proteins called SUN-domain proteins regulates a range of nuclear activities at the nuclear periphery, including tethering the centrosomes to the nuclear membranes. Defective SUN-domain proteins have been linked to certain types of muscular dystrophy and premature aging in humans. In this study, we use budding yeast as a model and show that its sole SUN-domain protein, Mps3, a key component of the yeast centrosome, is phosphorylated and then subject to proteolytic cleavage in order to properly split the centrosomes before spindle formation and chromosome segregation. Understanding how SUN-domain proteins are posttranslationally modified can shed light on the regulation of centrosome separation; it also provides insight into the homeostasis of SUN-domain proteins that are associated with many human diseases of the nuclear envelope.

## Introduction

Centrosomes nucleate microtubules and form a bipolar spindle that separates chromosomes during cell division. Like DNA replication, centrosome duplication occurs only once per cell cycle. Duplicated centrosomes are tethered, and their timely separation ensures accurate chromosome segregation. Supernumerary centrosomes and the subsequent formation of aberrant spindles can lead to aneuploidy in humans [1, 2]. At the core of the animal centrosome lies a pair of centrioles, whose cleavage by the cysteine protease, separase, necessitates their disengagement [3], although the substrate of the separase at the centriole remains controversial. In contrast, how the centrosome linkage is dissolved is less clear. In particular whether irreversible protein cleavage is required for centrosome separation, also called centrosome disjunction in animal cells, is not known.

**Fig 1:**
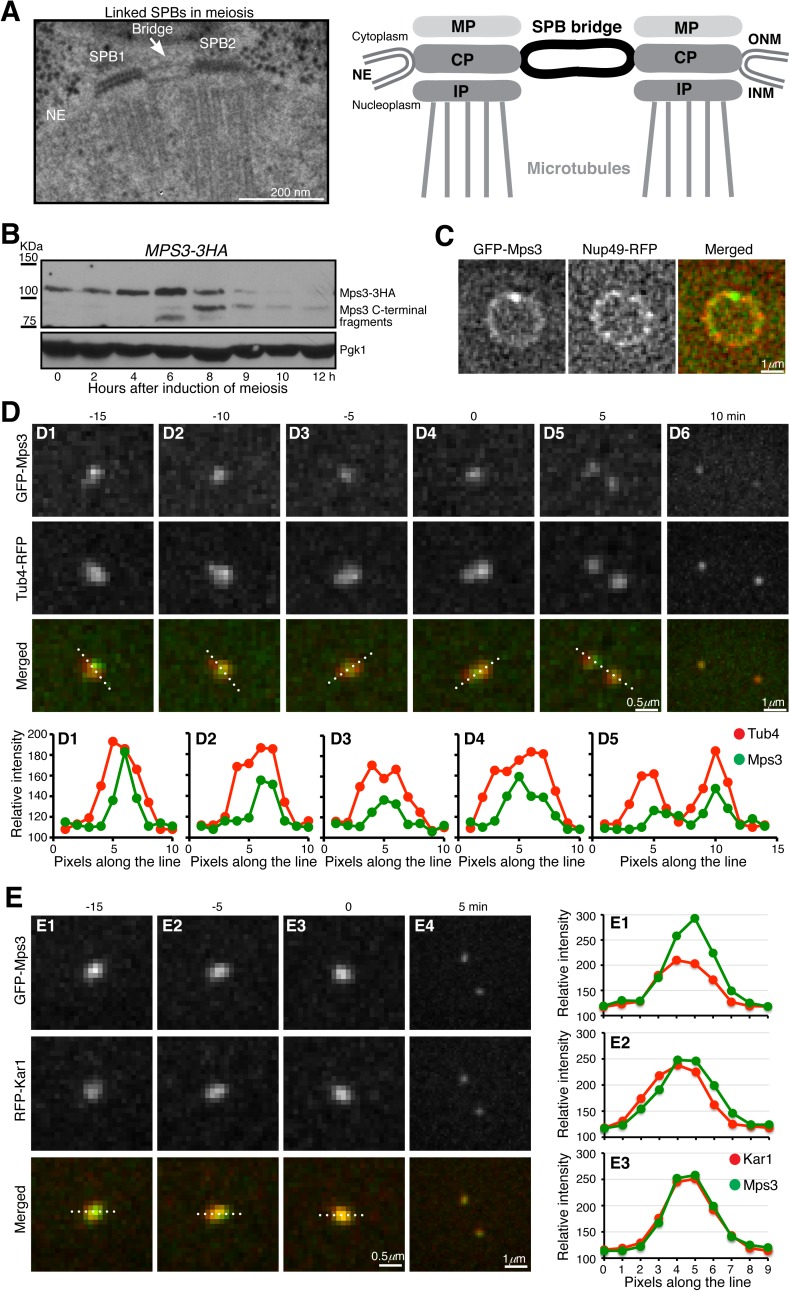
Localization of Mps3 during meiosis. (**A**) Model for linked SPBs. Left, an electron micrograph showing a pair of side-by-side SPBs in meiosis [21]. Right, a diagram depicting the bridge that connects SPBs. MP (meiotic), CP (central) and IP (inner) plaques of the SPB are shown. NE, nuclear envelope; ONM, outer nuclear membrane; INM, inner nuclear membrane. (**B**) Protein level of Mps3 during yeast meiosis. Yeast cells were induced to undergo synchronous meiosis, and cell aliquots were withdrawn at indicated times for western blot. Mps3 was tagged with the HA epitope at its C-terminus and detected by an anti-HA antibody (12CA5). The level of Pgk1 serves as a loading control. Strain HY3871 was used. (**C**) Localization of Mps3 to the nuclear periphery during meiosis. Nup49, a core component of the nuclear pore complex, was tagged with RFP at its C-terminus and serves as a marker for the nuclear envelope. Strain HY5277. (**D**) Localization of Mps3 to the SPB during meiosis. Yeast cells were induced to undergo synchronous meiosis for about 4 hours, and time-lapse fluorescence microscopy was performed to visualize the localization of GFP-Mps3 and Tub4-RFP. Tub4, the γ-tubulin in yeast, was tagged with RFP at its C-terminus and serves as a marker for the SPB. Time-lapse intervals were set at 5 minutes. Time zero is defined as the point of SPB separation. Projected images from 12 z-stacks are shown. Fluorescence intensity of line scans along the Tub4-RFP axis was plotted in the graphs shown at the bottom. Dashed lines indicate the position of line scan. The SPB axis changed due to SPB movement during the time course. Strain HY5033. (**E**) Localization of Mps3 and Kar1 during meiosis I. Time-lapse fluorescence microscopy was performed as shown in panel **D**. The expression of *GPF-MPS3* and *RFP-KAR1* was under the control of their endogenous promoters. Fluorescence intensity of line scans of GFP-Mps3 and RFP-Kar1 was plotted in the graphs shown to the right. Note that the Kar1 signal increased at t = 0, whereas the Mps3 signal decreased when compared to its intensity at earlier time points. Strain HY5464.

In yeast, the centrosome is often referred to as the spindle pole body (SPB), which shares structural components with and is functionally equivalent to the human centrosome [4, 5]. One unique feature in budding yeast is that the SPB is embedded in the nuclear envelope. Duplicated SPBs are tethered by a nuclear membrane-anchored structure called the half-bridge [6], two of which form the full bridge (Fig 1A). There are four known half-bridge components; three of them, Sfi1 (called hSfi1 in human) [5], Cdc31 (called centrin in animals)[7] and Kar1[8], localize to the cytoplasmic side of the bridge, whereas Mps3 localizes to the nuclear side [9, 10]. Currently unknown is whether protein cleavage is required for yeast centrosome separation. If so, what is the nature of the protease that cleaves the yeast centrosomes?

During vegetative growth, the yeast SPB is duplicated at late G_1_ phase or early S phase of the cell cycle, but duplicated SPBs separate immediately in order to form a bipolar spindle even when DNA is still being replicated [4, 11]. SPB separation requires kinase activities from the cell-cycle-dependent kinase Cdk1 and the polo-like kinase Cdc5 [12–14]. Recent studies have shown that Sfi1 is a target of both Cdk1 and Cdc5, and phosphorylation of Sfi1 plays a critical role in both SPB duplication and separation [15, 16]. The current model for SPB linkage posits that at the cytoplasmic side of the nuclear envelope, Sfi1 forms protein dimers that span the entire bridge to mediate SPB cohesion [17, 18]. Together with the actions from Cdc31 and Kar1, Sfi1 tethers duplicated SPBs to generate a side-by-side SPB configuration (Fig 1A). Phosphorylation of Sfi1 at its C-terminal domain plays a role in SPB separation [15, 16, 19]. But how Mps3 forms the half-bridge at the nuclear side of the membrane and what its contribution is in SPB separation both are unclear.

In contrast to their separation within minutes after duplication in mitosis, duplicated SPBs are linked for hours during the meiotic G_2_ phase (often called prophase I) when recombination takes place [20, 21]. The telomere-associated protein Ndj1 also localizes to the SPB in an Mps3-dependent manner and protects SPBs from premature separation during meiosis [22]. Ndj1 binds to the N-terminal region of Mps3 [22, 23], indicating that Mps3 is the target of Ndj1 at the SPB.

Mps3 belongs to the SUN-domain protein family, in which the SUN domain is typically located at the C-terminus of the protein and is found in the lumen of the nuclear envelope [9, 24]. The SUN domain of Mps3 is required for targeting Mps3 to the SPB and for inserting the newly duplicated SPB into the nuclear membranes [25, 26]. In contrast, the N-terminal region of Mps3, which is present in the nucleoplasm, appears dispensable in mitosis, because deletion of the first 64 amino acids of Mps3 does not interfere with cell growth [10]. However, during meiosis the N-terminal domain is required for Ndj1 binding and regulates telomere movement at the nuclear periphery [22, 23].

Here we investigate how Mps3, in particular its N-terminal domain, regulates SPB cohesion. We have determined that Mps3 is proteolytically cleaved at its N-terminal domain during yeast meiosis. Noncleavable Mps3 inhibits SPB separation, demonstrating that cleavage of Mps3 is a critical event in the process of SPB disengagement and spindle formation in budding yeast meiosis.

## Results

### Mps3 localizes to the SPB half-bridge area during yeast meiosis

We have shown previously that Mps3 is an abundant but unstable protein during yeast meiosis [22]. Using a C-terminally HA-tagged allele of *MPS3*, which was incorporated at the endogenous *MPS3* locus and served as the only functional copy of *MPS3* in the yeast genome, we found that the level of Mps3 protein peaked 6 h after meiotic induction (Fig 1B), the time of which corresponded to meiosis I in the SK1 yeast genetic background. The level of the full-length Mps3 then decreased as yeast cells exited meiosis I, with the concomitant appearance of a prominent C-terminal fragment, about 12 kDa smaller than the full length Mps3 (Fig 1B). Note that this C-terminal fragment of Mps3 persisted toward the end of meiosis and appeared to have a longer half-life than the full length Mps3 (Fig 1B and see below). These findings indicate that the Mps3 protein is subject to modification during yeast meiosis.

To determine the localization of Mps3 to the SPB, we used an N-terminally tagged *GFP-MPS3* allele, which also was the only functional copy of the *MPS3* gene in these cells. By fluorescence microscopy of live meiotic yeast cells, we found that Mps3 localized to the nuclear periphery, but it was concentrated at the SPB (Fig 1C and 1D). Line scan of the fluorescence intensities of Mps3 and the SPB marker Tub4, which was fused to RFP, revealed colocalization of Mps3 with Tub4 (Fig 1D). Crucially, ~10 min before SPB separation at the transition from prophase I to metaphase I when the Tub4 signal was stretched to form an axial line, the GFP-Mps3 signal remained focused and was found at the center of that of Tub4 (Fig 1D), demonstrating Mps3’s localization to the SPB half-bridge area as shown previously in mitosis [9, 27]. By fluorescence microscopy, we further observed that Mps3 colocalized with another half-bridge component, Kar1, during yeast meiosis (Fig 1E). Together, these observations support the notion that Mps3 preferentially localizes to the half-bridge area of the SPB.

### Mps3 is cleaved at its N-terminal domain

**Fig 2:**
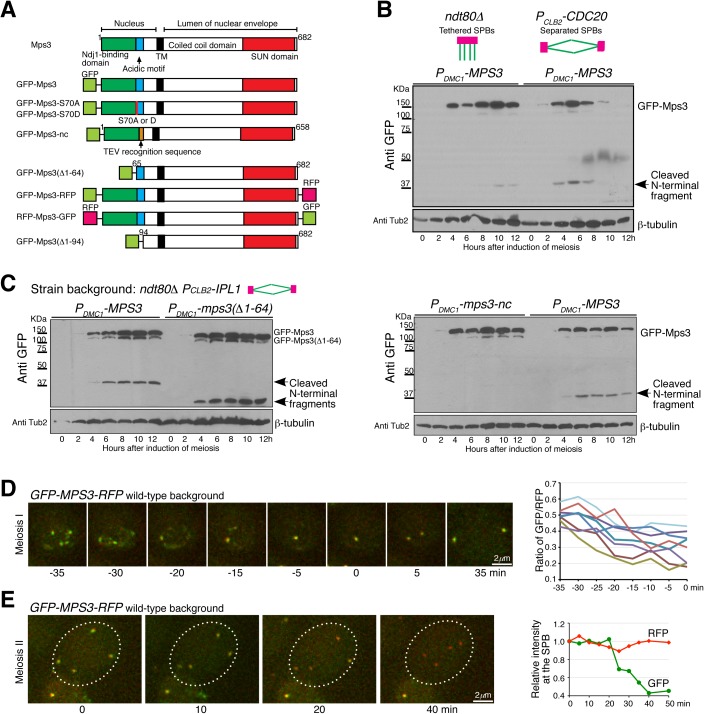
Cleavage of Mps3 during meiosis. (**A**) Schematic diagrams showing Mps3 protein structure and *MPS3* constructs used in this study. TM, transmembrane domain. (**B**) Mps3 protein level at prophase I and metaphase I. Yeast cells were induced to undergo synchronous meiosis, and cell aliquots were withdrawn at indicated times for western blot. GFP-Mps3 was detected by an anti-GFP antibody. The arrow points to the cleaved N-terminal fragment from Mps3. Strains HY4430 and HY5268. (**C**) Western blots probing Mps3 cleavage during meiosis as shown in panel **B**. Note that removal of the first 64 amino acids from Mps3 reduced the size of GFP-Mps3 and its cleaved product. Strains HY4432 and HY4484. (**D**) Cytological observation of Mps3 cleavage at the SPB in meiosis I. Yeast cells were induced to undergo synchronous meiosis for about 4 h, and cell aliquots were prepared for time-lapse fluorescence microscopy. The ratio of RFP over GFP at the SPB from 8 cells (n = 26) is shown to the right. Time zero is defined as the point of SPB separation in meiosis I. Projected images from 12 z-stacks were used for display. Strain HY5098. (**E**) Cytological observation of Mps3 cleavage in meiosis II (n = 50). The averaged fluorescence intensity of RFP and GFP at the SPBs from the cell shown to the left is plotted over time. Time zero is defined as the start point of microscopy. Strain HY5098.

We noticed that the fluorescence intensity of GFP-Mps3 decreased at the SPB prior to SPB separation at metaphase I (Fig 1D and 1E). This was in contrast to the fluorescence of Kar1, which was fused with the RFP at its N-terminus and reached its highest intensity at the time of SPB separation (Fig 1E). To produce the Mps3 protein more abundantly during meiosis for western blot analysis of its N-terminus, we used the meiosis-specific *DMC1* promoter to induce *MPS3* expression (Fig 2B and 2C). Note that in these cells, the endogenous copy of *MPS3* was present, because *MPS3* is an essential gene for cell viability. The level of the full-length Mps3 remained relatively stable when yeast cells were arrested at prophase I by means of deletion of *NDT80* (Fig 2A and 2B), which encodes a meiosis-specific transcription factor that is required for mid and late meiotic gene expression[28]. In contrast, the level of Mps3 fell precipitously when cells transited from prophase I to metaphase I as shown in Cdc20-depleted (*P*_*CLB2*_*-CDC20)* cells (Fig 2B), indicating that degradation of Mps3 occurs at the time of SPB separation.

We seek to understand how Mps3 is degraded. By western blotting, we observed the formation of an N-terminal fragment of Mps3 at the molecular weight of ~12 kDa, excluding the GFP tag, in meiotic cells (Fig 2B and 2C and S1A Fig). The abundance of this fragment peaked 6 h after the induction of meiosis in the wild-type background (S1A Fig), the time which corresponds to meiosis I SPB segregation [22]. In contrast, the N-terminal fragment was present at a much lower level, and its appearance was delayed in *ndt80Δ* cells arrested at prophase I (Fig 2B). To determine the biological relevance of the formation of this N-terminal fragment, we staged yeast cells at prophase I using the *ndt80Δ* allele and simultaneously depleted meiotic Ipl1p (*P*_*CLB2*_*-IPL1*), because in double *ipl1 ndt80Δ* mutant cells, SPBs can separate prematurely at prophase I without the activation of the mitotic cyclin-Cdk1 [21, 29, 30]. Crucially, in the absence of Ipl1, the N-terminal fragment of Mps3 became abundant at prophase I (Fig 2C). These results support the idea that Mps3 is cleaved at its N-terminus, the process of which appears to be correlated with SPB separation during meiosis I in budding yeast.

To test the hypothesis that Mps3 was cleaved prior to SPB separation, first we deleted the N-terminal 64 amino acids from Mps3 because removal of these residues abolishes Ndj1’s binding to Mps3 [22, 23]. The size of the N-terminal fragment became correspondingly smaller without the first 64 residues (Fig 2C), suggesting a site-specific cleavage event occurred beyond position 64. Next, on the basis of the molecular weight of the cleaved product, we replaced amino acids 65 to 93 of Mps3 with the TEV protease recognition sequence to generate *mps3-nc*, which effectively abolished the formation of the N-terminal fragment (Fig 2A and 2C and see below). Amino acid 93 ends at the acidic motif of Mps3 that has been described previously [25]. The above findings are consistent with the notion that Mps3 is cleaved specifically at its N-terminal domain, which binds to Ndj1 and is located in the nucleoplasma.

### Cleavage of Mps3 takes place at the SPB and at the nuclear periphery

To determine whether Mps3 was cleaved at the SPB, we constructed a *GFP-MPS3-RFP* double-tagged allele (Fig 2A), which was under the control of the endogenous *MPS3* promoter and the only functional copy of *MPS3* in these cells. By time-lapse fluorescence microscopy, we observed cells that were undergoing prophase I to metaphase I transition, at which point Mps3 formed a distinctive focus at the SPB (Fig 2D). The ratio of the fluorescence intensity of RFP, fused to the C-terminus of Mps3, over that of GFP, fused to the N-terminus, increased up to 50% prior to SPB separation (Fig 2D), indicating that roughly a third of the SPB-associated Mps3 is cleaved at its N-terminal domain. This estimate likely underrepresents the real rate of N-terminal cleavage, because reloading of the full length Mps3 at the SPB appeared active before SPB reduplication at interphase II (Fig 2D and see below). At the end of meiosis II, majority of Mps3 was cleaved at the N-terminal domain, leaving only the C-terminal RFP-fused Mps3 fragment visible (Fig 2E). Of note, the cleaved C-terminal fragment of Mps3, which is expected to retain its transmembrane domain and therefore remains anchored at the nuclear periphery, has a longer half life than the cleaved N-terminal fragment, making it possible for us to directly visualize Mps3 cleavage in live meiotic yeast cells by fluorescence microscopy. The above results also indicate that cleavage of Mps3 appears to take place throughout meiosis, and therefore meiosis II cells can serve as a reliable cytological readout of Mps3 cleavage.

As a control, we swapped the tags to construct an *RFP-MPS3-GFP* allele, which was also under the control of its endogenous promoter and the only functional copy of *MPS3* in these cells (S2 Fig). For the same reason, the full length of the Mps3 protein appeared continuously produced, then reloaded at the SPB during meiosis I. We therefore focused on cells in meiosis II when Mps3 was no longer produced, and found that in these cells, the GFP signal but not the RFP was retained much longer at the SPBs (S2 Fig). Combining the above biochemical and cytological findings, we conclude that the N-terminal domain of Mps3 is cleaved at the SPB during yeast meiosis.

**Fig 3:**
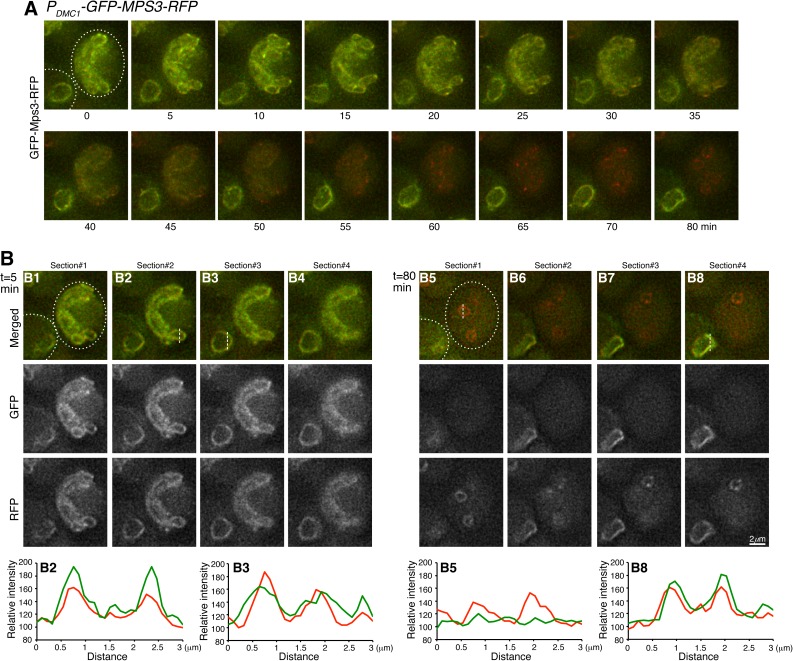
Cytological observation of Mps3 cleavage at the nuclear periphery. (**A**) Time-lapse microscopy showing the cell to the right of the field progressing through meiosis II. The expression of *GFP-MPS3-RFP* was under the control of the meiosis specific promoter *P*_*DMC1*_, which moderately overproduced Mps3. Projected images of 12 z-stacks are shown. Note that the right cell gradually lost its GFP signal, whereas much of the RFP signal remained at the nuclear periphery. The cell to the lower left corner remained at prophase I without nuclear division during the entire time course and serves as a control. Dashed lines depict the cell outline. (**B**) Selected single optical sections from t = 5 min and t = 80 min showing the signals of GFP and RFP from Mps3 as shown in panel **A**. Fluorescence intensity of line scans was plotted in the corresponding graphs shown at the bottom. The lower left cell (B3 and B8) serves as a control. Red, RFP; Green, GFP. Strain HY4835.

To determine whether membrane-bound Mps3, in addition to SPB-associated Mps3, was also cleaved at the nuclear periphery, we used the meiosis-specific *DMC1* promoter to express *GFP-MPS3-RFP* more abundantly (Fig 3A). Overproduced Mps3 protein localized robustly both to the SPB and to the nuclear periphery in meiosis I (Fig 3), demonstrating cytologically that the heterologous *P*_*DMC1*_*-MPS3* construct overexpressed *MPS3* during meiosis, as shown by western blotting (Fig 2B and 2C). To observe Mps3 cleavage, we focused again on cells in meiosis II when both the expression of *P*_*DMC1*_*-MPS3* and the production of Mps3 protein were terminated, permitting us to follow the fate of existing GFP-Mps3-RFP. Using time-lapse microscopy, we found that the RFP signal was retained much longer at the nuclear periphery at the end of meiosis (Fig 3A and 3B), indicating that the GFP fused to the N-terminus was removed earlier than the RFP fused to the C-terminus of Mps3. Therefore, Mps3 proteins located at the inner nuclear membrane are also cleaved during meiosis, just as are the ones associated with the SPB. Taking these observations together, we conclude that Mps3 is cleaved at its N-terminal domain, and that its cleavage can take place both at the SPB and at the nuclear periphery.

### Phosphorylation of Mps3 at S70 enhances Mps3 cleavage

**Fig 4:**
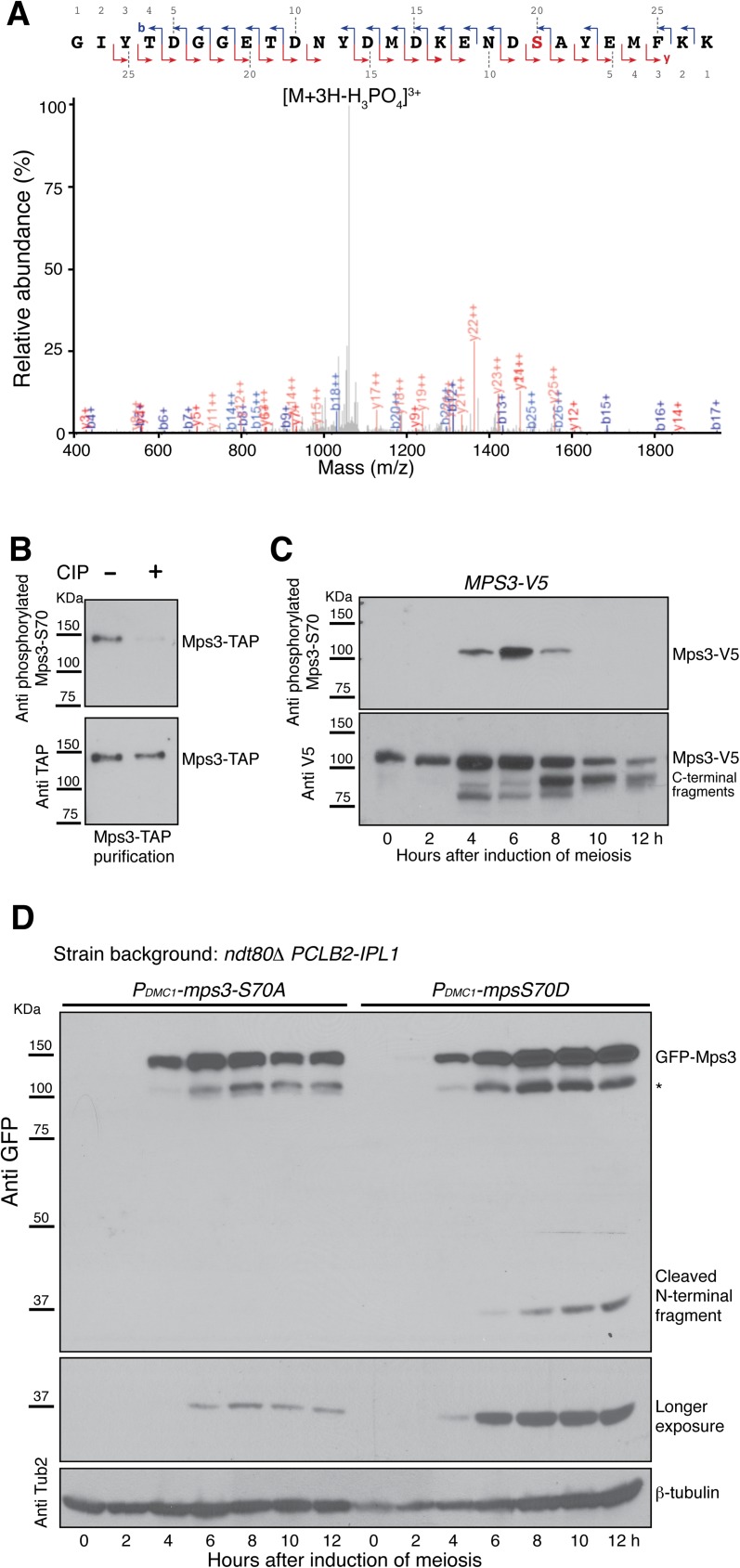
Phosphorylation at serine 70 promotes Mps3 cleavage. (**A**) Mass-spectrometry analysis of Mps3 phosphorylation. Yeast SPBs were affinity purified with Spc97-TAP, phosphopeptides were enriched and subjected to mass spectrometry. Mass to charge ratio (m/z) is shown at the x axis. The fragmentation map is shown at the top. Singly charged b-ions are shown in blue, y-ions in red, and doubly and triply charged ions in grey. Strain HY3674. (**B**) Mps3 is phosphorylated at S70 during yeast meiosis. Mps3-TAP was affinity purified from meiotic yeast cells and treated with the alkaline phosphatase (CIP). A phosphospecific antibody was used to determine phosphorylation at S70 of Mps3. Strain HY3810. (**C**) The timing of Mps3-S70 phosphorylation during meiosis. Yeast cells were induced to undergo synchronous meiosis, and protein extracts were prepared for western blotting. Note that Mps3 is highly phosphorylated at S70 6 h after the induction of meiosis. Strain HY5568. (**D**) Effect of Mps3 phosphorylation on Mps3 cleavage. Yeast cells were induced to undergo synchronous meiosis, and western blots were performed to probe the presence of the N-terminal fragment resulted from Mps3 cleavage. Note that Mps3-S70A, but not Mps3-S70D, inhibited Mps3 cleavage. * Degradation product of GFP-Mps3. Strains HY4431 and HY4468.

Using a protein mass spectrometry-based approach [31], we determined that Mps3 is phosphorylated at serine 70 during meiosis (Fig 4A). As we have shown previously, we used TAP tagged Spc97, a component of the γ-tubulin ring complex of the yeast SPB, to purify SPBs from yeast cells that were induced to undergo synchronous meiosis [22]; the enriched SPBs were then subject to SPB phosphoproteome (see materials and methods). By protein mass spectrometry, 38% of the Mps3 protein sequence was covered with a total of 39 spectra, of which 4 showed Mps3 phosphorylation at S70 (Fig 4A shows one of them). Because the Mps3 protein analyzed was copurified with Spc97-TAP, we conclude that SPB-associated Mps3 is phosphorylated at S70.

To start to determine how Mps3-S70 is phosphorylated during yeast meiosis, we raised a phosphospecific antibody against the phosphorylated form of Mps3-S70 (Fig 4B). Alkaline phosphatase treatment of affinity-purified Mps3-TAP extracted from meiotic yeast cells removed Mps3-S70 phosphorylation (Fig 4B), demonstrating the specificity of our antibody and further confirming the phosphorylation of Mps3 at S70 (Fig 4B). To determine the timing of Mps3-S70 phosphorylation, we performed western blotting on protein extracts from progressing meiotic yeast cells, of which the endogenous Mps3 was tagged with the V5 epitope at its C-terminus (Fig 1B). As shown in Fig 4C, Mps3-S70 was highly phosphorylated 6 h after induction of meiosis, the time which roughly corresponds to prophase I to metaphase I transition and SPB separation. We also observed Mps3-S70 phosphorylation in cells that were arrested at prophase I by *ndt80Δ* (S3 Fig), indicating that phosphorylation of Mps3 takes place at meiotic prophase I.

To determine whether S70 phosphorylation regulates Mps3 cleavage, we generated alleles of *mps3-S70A*, which abolished phosphorylation at position 70, and *mps3-S70D*, which mimicked phosphorylation at this position (Fig 2A). Both the *mps3-S70A* and *mps3-S70D* constructs were under the control of the *DMC1* promoter for expression during meiosis (Fig 4C). As shown in Fig 4D, the level of the cleaved N-terminal product was dramatically reduced in *mps3-S70A* cells, compared to that of *mps3-S70D* cells. Therefore phosphorylation of Mps3 at S70 increases the efficiency of its cleavage during meiosis.

### Cleavage of Mps3 depends on the proteasome activity

**Fig 5:**
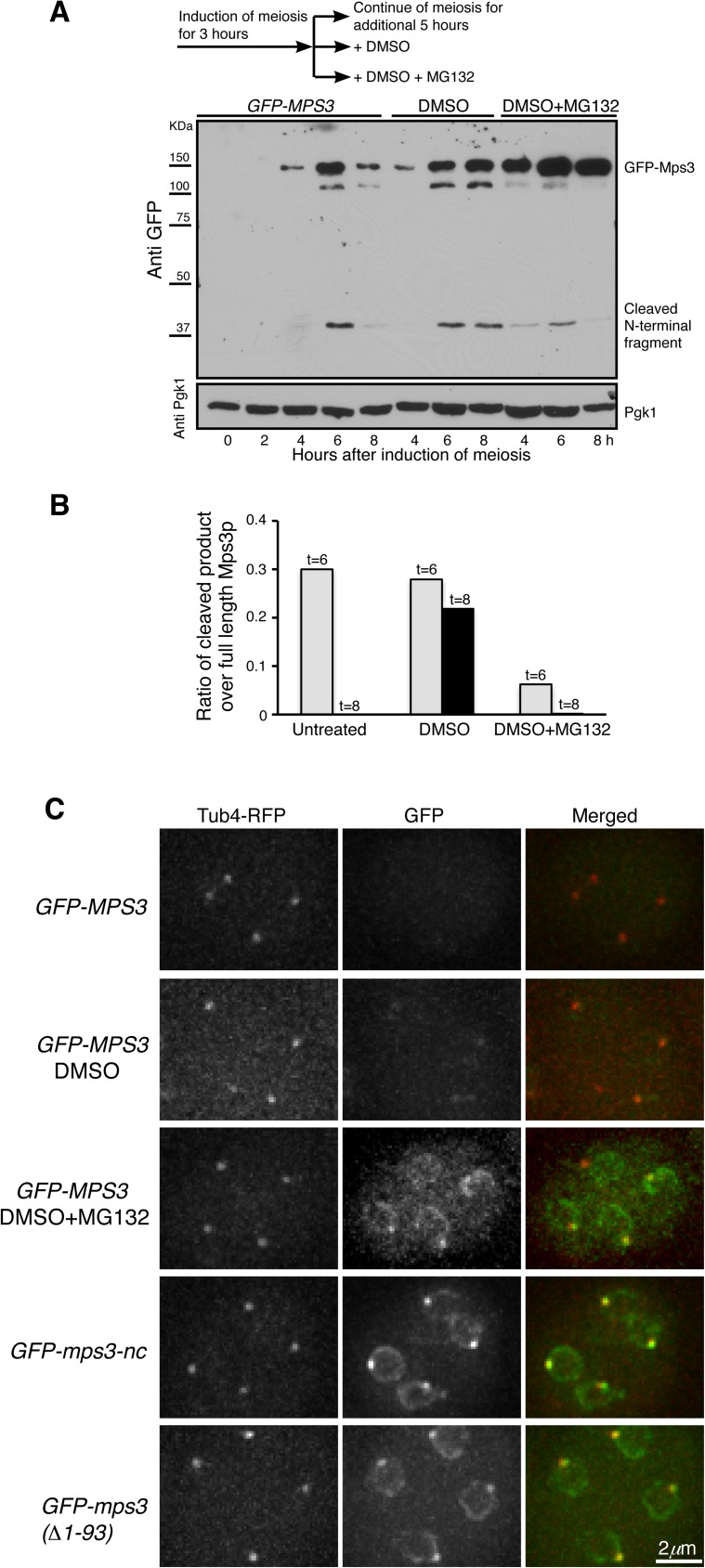
Proteasome-dependent cleavage of Mps3 at its N-terminal domain. (**A**) The proteasome activity is required for Mps3 cleavage. Yeast cells were induced to undergo synchronous meiosis for 3 h, and then the culture was split into three fractions as shown in the diagram on the top of this panel. Protein extracts were prepared from cells at indicated times for western blot and probed by an anti-GFP antibody. Note that the addition of MG132, but not DMSO, inhibited Mps3 cleavage. Strain HY4430. (**B**) Quantification of Mps3 cleavage in cells as shown in A. Gray bars show the values obtained 6 h after induction of meiosis; black bars show 8 h in DMSO and DMSO+MG132 treated samples. (**C**) Cytological evidence of Mps3 cleavage during yeast meiosis. Yeast cells were induced to undergo meiosis for about 12 h, and fluorescence microscopy was performed to observe GFP-Mps3 and Tub4-RFP localization in cells that had completed meiosis. Projected images from 12 z-stacks were shown. Tub4-RFP marks the SPB. In these strains, GFP was fused to the N-terminus of Mps3. The *GFP-MPS3* constructs were under the control of the *DMC1* promoter. Note that treatment of cells with MG132 inhibited Mps3 cleavage in wild-type cells, whereas *mps3-nc* and *mps3(Δ1–93)* cells lacked cleavage. Strains HY4430, HY4373 and HY4978.

In our search for the protease that is responsible for Mps3 cleavage, we hypothesized that the proteasome regulates Mps3 cleavage because previous work has shown the specific regulation of proteasome activity during yeast meiosis [32, 33]. To inactivate the proteasome, we induced yeast cells to undergo synchronous meiosis and 3 h later added MG132, a potent inhibitor of the proteasome, to the yeast culture medium (Fig 5A diagram). In normally progressing meiotic cells, the cleaved Mps3 N-terminal fragment appeared most abundantly 6 h after the induction of meiosis (Fig 5A). In contrast, addition of MG132 greatly inhibited the production of the Mps3 N-terminal fragment, indicating that Mps3 cleavage is regulated by the proteasome activity (Fig 5A and 5B). Of note, in cells treated with MG132, Mps3 protein degradation was also inhibited, resulting in the increased level of the full length Mps3 (Fig 5A). Using an alternative genetic approach to inactivate the proteasome, we deleted *PRE9*, which encodes the nonessential α3 subunit of the 20S proteasome [34]. In the absence of α3, the α4 subunit can take over α3’s place to form a functional proteasome [34], but the activity of this α3-less proteasome is impaired at an elevated temperature [35]. In wild-type cells that were induced to undergo meiosis at 33°C, we found that Mps3 was cleaved just as those undergoing meiosis at 30°C (Fig 2B and S1A Fig), the optimal temperature for yeast sporulation. In contrast, cleavage of Mps3 was inhibited in *pre9Δ* cells as determined by western blotting (S1A Fig), further demonstrating that the proteasome activity is required for Mps3 cleavage.

By fluorescence microscopy, we found that in both MG132-treated and *pre9Δ* cells, noncleaved Mps3 with intact N-terminus remained at the nuclear periphery and the SPBs at the end of meiosis, just as it was found in *mps3-nc* cells (Fig 5C and S1B Fig), demonstrating that Mps3 cleavage was inhibited in these cells. Taking these findings together, we conclude that the proteasome activity regulates Mps3 cleavage during yeast meiosis.

### Cleavage of Mps3 promotes SPB separation

**Fig 6:**
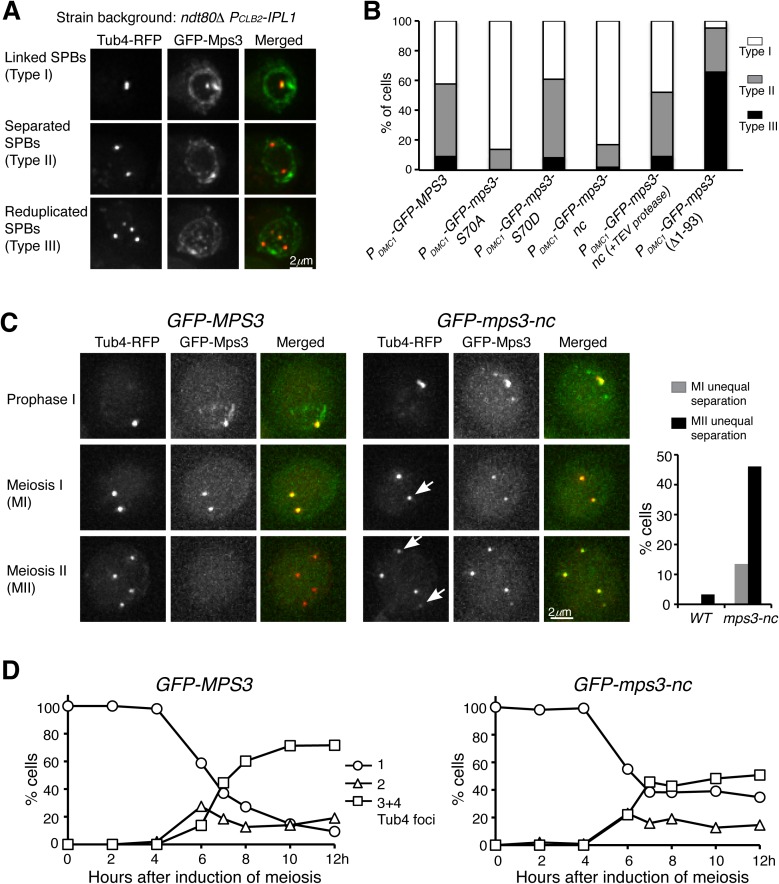
The role of Mps3 cleavage in SPB separation. (**A**) Representative images showing SPB separation at prophase I. Three categories of SPB states were classified: Type I, SPBs remained cohesive; Type II, SPBs separated once; Type III, SPBs were reduplicated and separated twice. (**B**) Characterization of SPB separation in *mps3* mutants. The expression of GFP-tagged constructs and the TEV protease were under the control of the *P*_*DMC1*_ promoter. The wild-type copy of *MPS3* was presented in these cells. About 200 cells were counted for each strain 10 h after the induction of meiosis. The averages of more than two independent experiments are shown. Strains HY4430, HY4431, HY4432, HY4468, HY4824 and HY4970. (**C**) Representative images showing SPB separation during meiosis. Yeast cells were induced to undergo synchronous meiosis, and live-cell fluorescence microscopy was performed to determine the localization of GFP-Mps3, GFP-Mps3-nc and Tub4-RFP. Both *GFP-MPS3* and *GFP-mps3-nc* were under the control of the endogenous MPS3 promoter. Projected images are shown. Arrows indicate unequal SPB separation in *mps3-nc* cells. The graph to the right shows the percentage of cells showing unequal SPB separation in meiosis I (gray bars) and meiosis II (dark bars). (**D**) Quantification of SPB separation as shown in C. About 200 cells were counted at each time point. The averages of two independent experiments are shown. Strains HY5741 and HY5742.

Because the timing of Mps3 cleavage correlates to that of SPB separation in meiosis I, we hypothesized that cleavage of Mps3 was necessary for SPB separation. Previously we have shown that depletion of Ipl1 during meiosis can lead to premature SPB separation at prophase I; we therefore used the *ipl1 ndt80Δ* strain as a tool to assay SPB separation (Fig 6A). Because *MPS3* is an essential gene, we overexpressed GFP-tagged *MPS3* alleles specifically in meiosis with the heterologous *P*_*DMC1*_*-GFP-MPS3* expression constructs (Fig 6A and 6B). As such, any *mps3* mutant phenotype observed would be dominant negative. When the wild-type *GFP-MPS3* allele was expressed, about 58% of the cells separated SPBs 10 h after the initiation of meiosis; among them, 9% had completed the second round of SPB duplication and separation (Fig 6B). Abolishing S70 phosphorylation (*mps3-S70A*) drastically inhibited SPB separation, whereas the *mps3-S70D* mutation retained the wild-type level of separation (Fig 6B). As expected, eliminating Mps3 cleavage in *mps3-nc* cells severely inhibited SPB separation (Fig 6B). Crucially introduction of the TEV protease into the *mps3-nc* cells restored SPB separation to the wild-type level (Fig 6B). Therefore both phosphorylation and cleavage of Mps3 are important for SPB separation in *ipl1 ndt80Δ* cells during meiosis.

If cleavage of Mps3 facilitates SPB separation, we expect that overexpression of Mps3(Δ1–93), which lacks the N-terminal domain, would lead to a higher level of SPB separation in the *ipl1 ndt80*Δ strain at prophase I. Indeed, 95% of *mps3(*Δ*1–93)* cells separated their SPBs prematurely, and more than 66% of them completed the second round of SPB duplication and separation in the *ipl1 ndt80*Δ background (Fig 6B). Because the Mps3(Δ1–93) protein lacked the cleavage site, N-terminal GFP tagged Mps3(Δ1–93) was found at the SPB and the nuclear membranes at the end of meiosis (Fig 5C). These findings support the notion that removal of the N-terminal domain from Mps3 promotes SPB separation.

To further determine the effect of Mps3 cleavage on SPB separation and cell cycle progression in meiosis, we generated an *mps3-nc* mutant allele that was under the control of its endogenous promoter and served as the only copy of the *MPS3* gene. In the absence of Mps3 cleavage, yeast cells largely retained viability during vegetative growth and were able to be induced to undergo meiosis (Fig 6C). Although early meiotic progression appeared to be on schedule in *mps3-nc* mutant cells, 35% of the mutant cells aborted meiosis without SPB separation and spore formation after 12 h in the sporulation medium, whereas more than 90% wild type cells formed tetrads or dyads at that time (Fig 6D). Importantly, among the mutant cells that underwent second round of SPB separation, 46% of them showed unequal SPB separation on the basis of the fluorescence intensity of Tub4-RFP and GFP-Mps3 (Fig 6C, arrows), indicating that noncleavable Mps3 inhibits proper SPB disjunction during meiosis. These observations demonstrate an essential role of Mps3 cleavage in SPB separation and cell cycle progression during yeast meiosis.

### Overexpression of Mps3-nc inhibits SPB separation and is lethal in vegetative yeast cells

**Fig 7:**
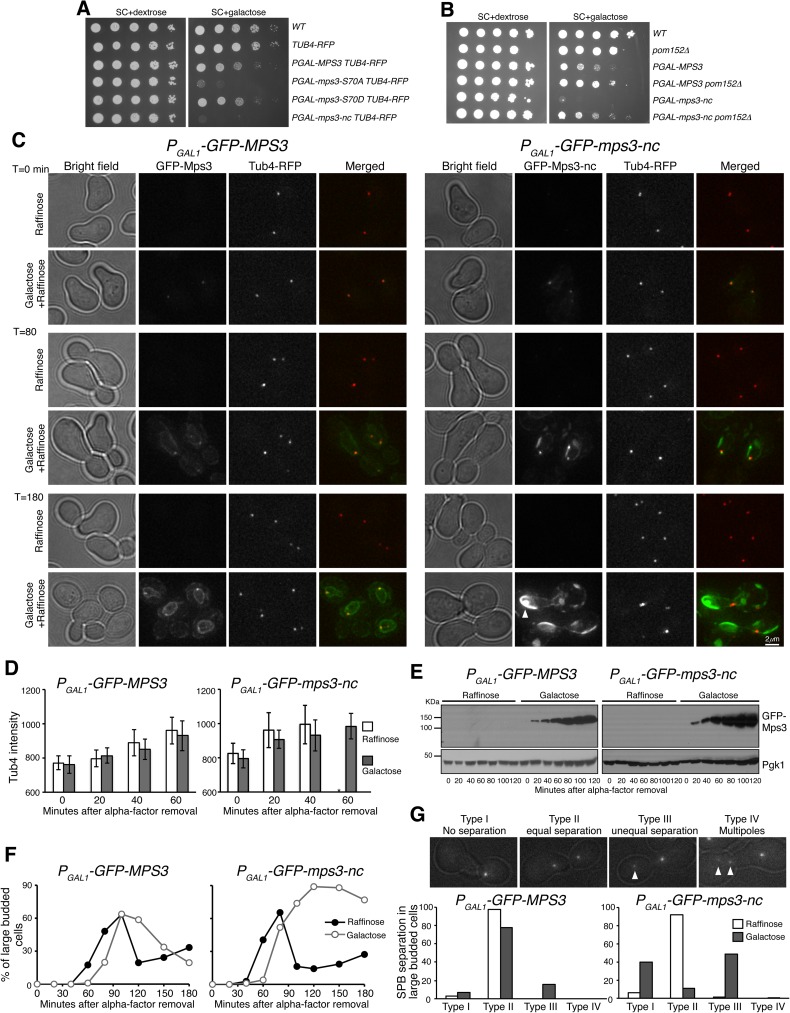
Phenotypes of *MPS3* overexpression and inhibition of SPB separation by noncleavable Mps3 in mitosis. (**A**) The effect of overexpression of *mps3-S70A* and *mps3-nc* on cell growth. Yeast strains were grown in YPD to saturation, tenfold diluted, and spotted onto SC+dextrose and SC+galactose plates. (**B**) Genetic interaction between *mps3-nc* and *pom152*Δ. Tenfold serial dilutions of yeast cells were spotted onto SC+dextrose and SC+galactose plates as shown in panel **A**. Note that *pom152*Δ partially suppressed the lethality of *P*_*GAL1*_*-mps3-nc* expression, indicating that the toxicity caused by *mps3-nc* originated at the yeast centrosome. (**C**) Accumulation of noncleavable Mps3 at the nuclear periphery. Yeast cells were grown in raffinose medium and arrested at G_1_ with the alpha-factor; expression of the *GAL1* promoter was then induced by the addition of galactose 30 min before removal of alpha-factor. Aliquots were withdrawn at indicated times and prepared for live-cell fluorescence microscopy. Time zero is defined as the point of alpha-factor removal. Representative images from wild-type and *mps3-nc* cells are shown. Tub4-RFP marks the SPB. Note that SPBs failed to separate or misseparated in cells with GFP-Mps3-nc. GFP-Mps3-nc first localized to the SPB, and then accumulated at the nuclear periphery, forming protein aggregates in large budded cells (arrows). In contrast, wild-type GFP-Mps3 was concentrated at the SPB and localized evenly around the nuclear envelope. (**D**) Quantification of Tub4-RFP fluorescence intensity before SPB separation. Error bars represent SD. * Wild-type cells separated their SPBs at t = 60 min when grown in raffinose. (**E**) Protein levels of Mps3 and Mps3-nc during mitosis. Cell aliquots were withdrawn at indicated times, and were prepared for western blot. The level of Pgk1 serves as a loading control. (**F**) Budding index of wild-type and *mps3-nc* cells during mitosis. Cell aliquots were withdrawn at indicated times, and budding morphology was determined by phase contrast microscopy. Strains HY4365 and HY5384. (**G**) Quantification of SPB separation. Yeast cells were withdrawn and prepared for live-cell microscopy. Four categories of SPB separation in large budded cells were classified: type I, one Tub4 dot (duplicated SPBs remain linked); type II, two equal Tub4 dots (equal SPB separation); type III, two unequal Tub4 dots (unequal SPB separation); type IV, more than two Tub4 dots (multiple spindle pole formation). Arrows point to examples of unequal SPB separation. Two independent experiments of alpha-factor arrest and release were performed.

To determine whether posttranslational modification of Mps3 was necessary for SPB separation during mitosis, we expressed *MPS3* mutants in vegetative yeast cells from the S288C background using a galactose inducible promoter (Fig 7 and S4 Fig). Overexpression of *mps3-nc* was lethal to yeast cells (Fig 7A), but this lethal phenotype could be suppressed by *pom152*Δ (Fig 7B), indicating that the lethality caused by *mps3-nc* is due to the mitotic defects occurring at the SPB, because *pom152*Δ restores proper SPB function when Mps3 is absent [36]. To pinpoint the role of Mps3 in SPB separation, we synchronized yeast cells at G_1_, induced *MPS3* expression, and then released these cells from G_1_ arrest to analyze SPB duplication and separation (Fig 7C). Galactose-induced Mps3, including Mps3-nc, first appeared at the SPB (Fig 7C). Notably, Mps3-nc started to form protein aggregates at the nuclear periphery about 80 min after the activation of the galactose promoter (Fig 7C). On the basis of the fluorescence intensity of Tub4-RFP, SPB duplication appeared comparable between wild-type and *mps3-nc* cells (Fig 7D), and wild-type and mutant proteins were produced in a similar time frame (Fig 7E). However, cells expressing *mps3-nc* were essentially blocked at anaphase with large buds (Fig 7F). In large-budded cells that overexpressed *mps3-nc*, 40% failed to separate their SPBs, and if they were separated, 49% showed unequal SPB separation (Fig 7G), demonstrating that *mps3-nc* cells either failed to separate or misseparated their SPBs. Failure to properly partition SPBs was also observed in cells overexpressing *mps3-S70A* (S4 Fig). We note that, under the control of its endogenous promoter, *mps3-nc* cells are viable, albeit with a slight growth defect. Together, our findings indicate that posttranslational modification of Mps3 is also critical for proper SPB separation in mitosis.

### Mps3 forms protein oligomers in vivo

**Fig 8:**
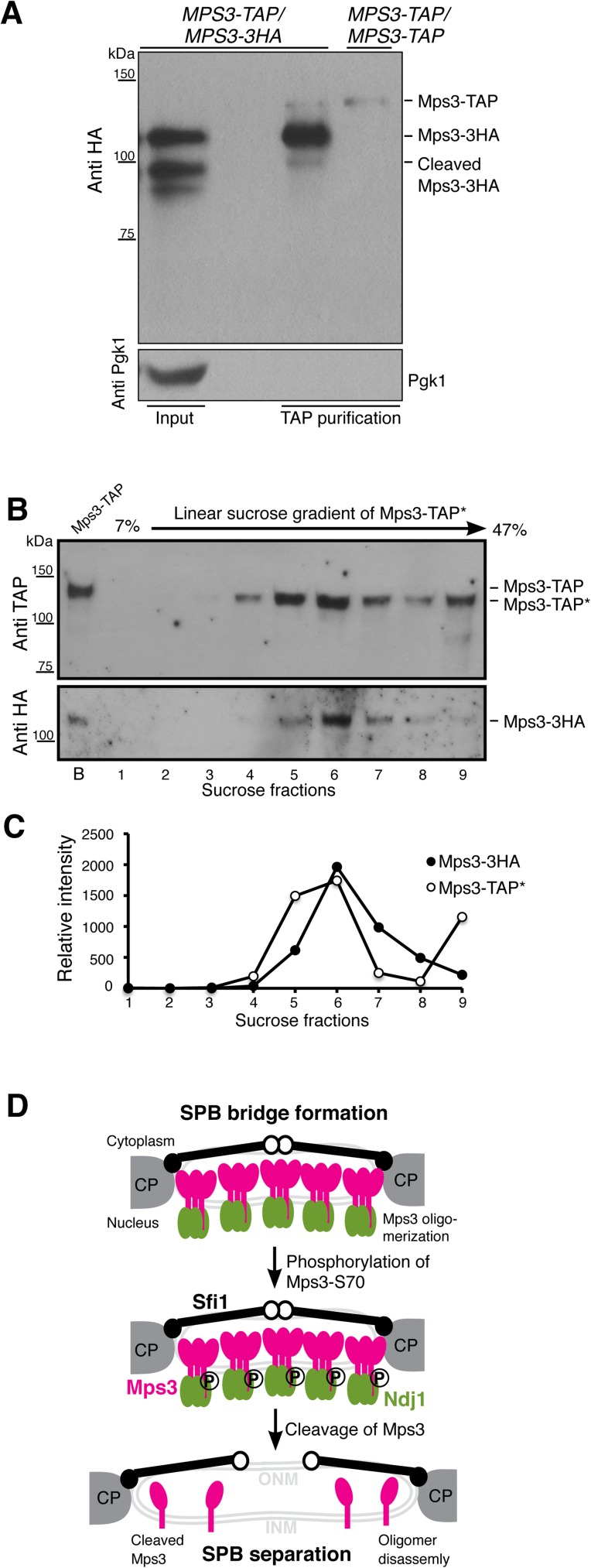
Model for Mps3 action during SPB separation. (**A**) Affinity protein purification showing that Mps3 forms protein oligomers during yeast meiosis. In the diploid yeast cell (HY4394), one copy of *MPS3* was tagged with TAP, the homologous copy was tagged with 3xHA, both to the C-terminus of Mps3. Yeast cells were induced to undergo synchronous meiosis for 5 h, and 2 liters of cells were harvested for protein affinity purification, followed by western blotting. Note that the full-length Mps3 minimally interacts with its cleaved form. Because the secondary antibody also recognizes the protein A peptide domain of the Mps3-TAP, here the *MPS3-TAP/MPS3-TAP* strain (HY3810) serves as a control to show the position of Mps3-TAP on the western blot. (**B** and **C**) TEV-cleaved Mps3-TAP* and Mps3-3HA peaked in the same sucrose fraction. Mps3-TAP was affinity purified as in A, followed by TEV protease treatment to remove the protein A peptide domain, thus releasing Mps3-TAP* from the magnetic beads. Affinity purified protein complexes were then fractionated by centrifugation in a linear sucrose gradient. Western blotting was used to determine the presence of Mps3-TAP* and Mps3-3HA in sucrose fractions. TAP* denotes the removal of the protein A domain from the TAP tag, thus Mps3-TAP* by itself can no longer be recognized by the secondary antibody. Lane B denotes bead-bound Mps3-TAP before TEV treatment, which has a higher molecular weight than TEV-cleaved Mps3-TAP*. Quantification of signal intensity of Mps3-TAP* and Mps3-3HA was shown in C. (**D**) Model for Mps3 cleavage and SPB separation. Mps3 is shown as red lollipops, Ndj1 as green ovals, and Sfi1 as black lines with a closed (N-terminus) and an open (C-terminus) dot. Part of the central plaque (CP) is shown in the diagrams of the SPB half-bridge.

The topology of Mps3 resembles a single-pass cell-surface receptor, which forms protein oligomers, typically dimers, upon ligand binding [37]. To determine whether Mps3 forms protein oligomers, we constructed an *MPS3-TAP/MPS3-3HA* strain, induced cells to undergo synchronous meiosis and performed TAP protein affinity purification to determine the physical interaction between Mps3-TAP and Mps3-3HA (Fig 8A–8C). Note that the tagged alleles of *MPS3* were incorporated at the endogenous *MPS3* locus and served as the only functional copies of *MPS3*. By western blotting, we found that Mps3-3HA was copurified with Mps3-TAP (Fig 8A), indicating that Mps3 can form homo oligomeric protein complexes in vivo. Crucially, Mps3-TAP appeared to interact more strongly with the full-length Mps3-3HA, but minimally with the N-terminally cleaved form of Mps3-3HA (Fig 8A and 8B), suggesting that without its N-terminal domain, Mps3 oligomer formation is weakened. This finding is consistent with the observation that *mps3(*Δ*1–93)* cells, which lacked the N-terminus domain of Mps3, separated their SPBs prematurely at prophase I (Fig 6B). In addition, we performed sucrose gradient fractionation of affinity purified Mps3 protein complexes and observed that Mps3-TAP*, of which the TAP tag was essentially removed enzymatically (see materials and methods), and Mps3-3HA peaked in the same sucrose fractions (Fig 8B and 8C), supporting the idea that Mps3 forms protein oligomers in vivo.

## Discussion

In this report, we have shown for the first time that a SUN-domain protein is cleaved at the nuclear membranes. Mps3 localizes to the inner nuclear membrane and to the nuclear side of the SPB half-bridge to mediate SPB cohesion, and its cleavage at the N-terminal domain plays a role in meiotic SPB separation. Posttranslational modifications, including protein phosphorylation [31], have been found to take place extensively at the yeast SPB, but irreversible protein cleavage at the half-bridge was not known previously. Our finding of Mps3 cleavage at the SPB and the nuclear membranes therefore provides insight into the mechanism of half-bridge disassembly, which is critical for proper centrosome separation and chromosome segregation.

Three lines of evidence we have obtained demonstrate that Mps3 is cleaved at its N-terminal domain, which is positioned in the nucleoplasm. First, our biochemical and genetic analysis reveals that Mps3 is cleaved at the acidic motif located at its N-terminal domain. Second, phosphorylation of serine 70, which is adjacent to the acidic motif, promotes Mps3 cleavage, perhaps by increasing the acidity or by another means of modifying the local environment that contains the cleavage site. Finally, our cytological observations unambiguously show that Mps3 can be cleaved at both the SPB and the nuclear periphery, providing strong evidence that the protease responsible for Mps3 cleavage is located at or adjacent to the inner nuclear membrane.

We propose that cleavage of Mps3, which removes the binding site for its nuclear ligands such as Ndj1, regulates Mps3 oligomer disassembly and half-bridge breakage (Fig 8D). Similar to a single-pass cell-surface receptor, upon which ligand binding on one side of the membrane often triggers conformational changes of the peptide domain that is located on the opposite side of the membrane [37], Mps3, when cleaved from its N-terminal domain, appears to have a reduced affinity for oligomer formation (this study). Our findings therefore indicate that the nucleoplasm-localized N-terminal domain of Mps3 has a profound impact on the conformation of its C-terminus, which is positioned in the lumen of the nuclear envelope and contains a coil-coiled segment and the SUN-domain, both of which have been implicated in protein trimerization [38–40]. Our findings also suggest that the bridge which tethers duplicated SPBs is disassembled, at least partially, at the point of SPB separation. Phosphorylation at the cytoplasmic side of the half-bridge, which is a reversible process, also regulates SPB separation [15, 16]. Therefore, posttranslational modifications of half-bridge components on both sides of the nuclear envelope appear to work either simultaneously or redundantly to ensure the timely separation of yeast centrosomes. Although the cyclin-dependent kinase Cdk1 and the polo kinase Cdc5 both have been implicated in phosphorylating the half-bridge component Sfi1 [15, 16], the nature of the kinase responsible for Mps3-S70 phosphorylation remains to be determined. We also note that in the absence of Mps3 cleavage, to some degree duplicated SPBs can be separated albeit with many cells showing unequal partitioning of SPB components. Our observations therefore indicate that the cytoskeleton, for example microtubule-based, forces could overwhelm the structural integrity of the half-bridge during SPB separation.

Because cleavage of Mps3 depends on the proteasome activity, we speculate that the proteasome, which is highly concentrated in the yeast nucleus [41], acts as the protease responsible for Mps3 cleavage. The proteasome is capable of mediating site-directed peptide cleavages after either the acid or basic amino acid residues [42]. Alternatively, proteasome activity is required for activating the yet unidentified protease that is directly responsible for Mps3 cleavage. In either scenario, the proteasome would play a crucial role in modifying Mps3 located at both the SPB half-bridge and the nuclear membranes. Noncleavable Mps3 accumulates at the nuclear periphery (this study), indicating that in addition to Mps3 cleavage, the proteasome activity also regulates Mps3 protein homeostasis at the nuclear membranes.

Mps3 is highly expressed in meiosis and regulates SPB cohesion (this study) and telomere attachment to the nuclear periphery [23], which raises an intriguing question: Is Mps3 cleavage specific to yeast meiosis? Our preliminary study of Mps3 in vegetative yeast cells using the inducible galactose promoter failed to detect its N-terminal cleaved fragment (Fig 7E), owing to a much short half-life of the cleaved products during mitosis. Alternatively, perhaps a specialized modification of Mps3 occurs in yeast meiosis, at which point SPBs remains tethered and telomeres are clustered for an extended period of time. Indeed, the meiosis-specific telomere-associated protein Ndj1 binds specifically to the N-terminus of Mps3 [22, 23], and ectopic expression of *NDJ1* in vegetative yeast cells is lethal [22]. Upon the transition of prophase I to metaphase I during meiosis, Ndj1 is degraded [22] and concomitantly Mps3 is cleaved, which leads to irreversible structural modifications at the SPB half-bridge and the telomeres. Therefore, at the nuclear periphery a unique signal transduction cascade appears to be in place during meiosis to regulate Mps3 phosphorylation and protein cleavage. (Mps3 cleavage and cell cycle progression).

Although SUN-domain proteins vary at their N-termini [24], Mps3 shares a high degree of conservation with SUN1 from mammals, in particular at the N-terminus [9, 26]. Phosphorylation at the N-terminal domain has been previously reported for SUN1 from mammals and SUN-1 in *C*. *elegans* [43, 44]. Intriguingly, at the meiotic G_2_, SUN-1 shows decreased affinity to an antibody raised specifically against its N-terminus, a finding which has been interpreted as epitope masking [43]. Alternatively, SUN-1 could be cleaved at its N-terminus, similar to our observation of Mps3 cleavage during yeast meiosis. Before their separation, animal centrosomes are also attached to, although not embedded in, the nuclear envelope in a SUN-domain-protein-dependent manner [45]. Therefore, lessons learned from yeast centrosome separation mediated by irreversible protein cleavage have implications for understanding the regulation of centrosome disjunction and other SUN-domain protein-mediated nuclear activities in all eukaryotes.

## Materials and methods

### Yeast strains and plasmids

Yeast strains and plasmids used in this study are listed in S1 Table and S2 Table. We used a PCR-based method [46] to introduce protein tags to the C-terminus of Mps3, including alleles of *MPS3-V5*, *MPS3-3HA*, *MPS3-TAP*, *MPS3-GFP* and *MPS3-RFP*. Using a similar strategy, we constructed *NUP49-RFP*. Nup49 is a core component of the nuclear pore complex and serves as a marker for the nuclear envelope (Fig 1C). The alleles of *TUB4-RFP* and *SPC97-TAP* have been reported previously [22]. These tagged alleles are the only functional copies of the corresponding genes in the yeast genome. A similar PCR-based method was used to replace the *PRE9* open reading frame with the KanMX cassette. Correct transformations were confirmed by colony-based diagnostic PCR. PCR primers are listed in S3 Table. The following mutant alleles have been reported previously: *ndt80*Δ [28], *P*_*CLB2*_*-CDC20* [47], *P*_*CLB2*_*-IPL1* [21], *P*_*CLB2-*_*MPS3* [22], *pom152*Δ [36] and *pdr5*Δ.

We used the *GAL1* promoter to induce expression of *MPS3* and its mutant alleles with galactose, and the *DMC1* promoter to express *MPS3* and its mutant alleles in meiosis. Using pRS305 as the cloning vector, we constructed alleles of *P*_*GAL1*_*-GFP-MPS3* (pHG323) and *P*_*DMC1*_*-GFP-MPS3* (pHG350). Other *MPS3* alleles were derivatives of these two. These heterologous *MPS3* constructs were linearized by StuI and incorporated at the endogenous *MPS3* locus by yeast transformation. We used a PCR-based mutagenesis method to generate point mutations of *mps3-S70A* and *mps3-S70D*, deletion mutations of *mps3(*Δ*1–64*) and *mps3(*Δ*1–93*), and a sequence replacement mutation (*mps3-nc*), of which amino acids 65 to 93 of Mps3 were replaced by the Tobacco Etch Virus (TEV) recognition sequence ENLYFQG (Fig 2A). The expression of the TEV protease was under the control of the *DMC1* promoter (pHG394). We cloned a fragment of 1 kb DNA sequence in front of the *MPS3* open reading frame to serve as the *MPS3* promoter and constructed alleles of *P*_*MPS3*_*-GFP-MPS3* (pHG454) and *P*_*MPS3*_*-RFP-MPS3* (pHG468) using plasmid pRS306 as the backbone. With a similar approach, we constructed alleles of *P*_*KAR1*_*-GFP-KAR1* (pHG465) and *P*_*KAR1*_*-RFP-KAR1* (pHG501). About 1 kb DNA sequence upstream of the *KAR1* open reading frame was cloned to serve as the *KAR1* promoter. Plasmids were linearized before their transformation to yeast cells.

To construct the double-tagged alleles of *P*_*MPS3*_*-GFP-MPS3-RFP* and *P*_*MPS3*_*-RFP-MPS3-GFP* (Figs 2 and 3), plasmids pHG454 and pHG468 were linearized with the restriction enzyme AflII, which cuts at the *MPS3* promoter, and then were transformed into *MPS3-RFP* and *MPS3-GFP* yeast cells, respectively. Positive yeast transformants were patched onto the 5’-fluoroorotic acid medium to select recombinants that had excised the untagged copy of *MPS3*. The double-tagged alleles of *MPS3*, which served as the only functional copy of *MPS3* in the yeast genome, were determined by microscopy and colony PCR. A similar approach was used to construct *GFP-MPS3* and *GFP-mps3-nc* alleles under the control of the endogenous *MPS3* promoter (Fig 6C and 6D). Plasmids pHG454 and pHG459 were linearized with AflII, and yeast transformants were selected on 5’-fluoroorotic acid plates for recombinants, which were then confirmed by microscopy and colony PCR.

In cells where the heterologous *MPS3* construct was under the control of either the *DMC1* or *GAL1* promoter, the wild-type copy of *MPS3* was retained because *MPS3* is an essential gene. As such, the phenotypes of *mps3-nc*, *mps3-S70A* and *mps3(*Δ*1–93*) shown in Figs 6, 7 and S4 are dominant negative.

### Culture methods

Yeast cells were grown in YPD (1% yeast extract, 2% peptone and 2% dextrose) [48] at 30°C. To induce synchronous meiosis, YPD cultures were diluted with YPA (1% yeast extract, 2% peptone and 2% potassium acetate) to reach OD (optical density, λ = 600nm) of 0.2 and incubated at 30°C for about 14 hours to reach a final OD of 1.6. Then, yeast cells were washed once in water and resuspended in 2% potassium acetate, the point of which was defined as time zero after the induction of meiosis. Cell aliquots were withdrawn at indicated times for microscopy and protein extraction. The *pre9Δ* cells were grown in the YPA media at 25°C and induced to undergo meiosis at 33°C to inactive the proteasome (S1 Fig). To chemically inactivate the proteasome activity (Fig 5), yeast cells with *pdr5Δ* were induced to undergo synchronous meiosis for 3 hours; the culture was then split into three fractions: untreated, DMSO only and DMSO with MG132 (50 μM final concentration). Cells aliquots were withdrawn at indicated times, and protein extracts were prepared for western blotting.

For mitotic experiments shown in Figs 7 and S4, synthetic complete (SC) medium [48] with 2% raffinose was used. To arrest yeast cells at G_1,_ we grew them in the raffinose medium to reach OD of 0.4, then added 10 μg/ml alpha factor. Galactose (2% final concentration) was added to the culture medium to induce the expression of the *GAL1* promoter 30 minutes before the removal of the alpha factor, upon which cells resumed mitosis. To determine cell viability (Fig 7A), yeast cells were grown overnight in YPD liquid medium to reach saturation, 10 fold diluted, spotted onto SC plates with either 2% dextrose or 2% galactose, and then incubated at 30°C for about two days.

### Time-lapse fluorescence microscopy

Time-lapse live-cell fluorescence microscopy was carried out on a DeltaVision imaging system (GE Healthcare Life Sciences) at 30°C. We used a 60x (NA = 1.40) objective lens on an inverted microscope (IX-71, Olympus). Microscopic images were acquired with a CoolSNAP HQ2 CCD camera (Photometrics). Pixel size was set at 0.10700 μm. Time intervals were set at 2 to 5 minutes, and optical sections at 12, each with 0.25–0.5μm thickness. Ultra-high signal-to-background hard coated custom filter sets were used. For GFP, the excitation spectrum was at 470/40 nm, emission spectrum at 525/50 nm; for RFP, excitation was at 572/35, and emission at 632/60 nm. During microscopy, yeast cells were grown on agarose pads as described previously [21]. To minimize photo toxicity to the cells and photo bleaching to fluorophores, we used neutral density filters to limit excitation light to 32% or less of the normal output from the fluorescence illuminator. Exposure time was set at 0.1 second or less.

For time-course experiments shown in Figs 6, 7 and S4, at least two independent experiments were performed, and more than 100 cells were analyzed for each strain at each time point.

### Microscopy data analysis

Acquired microscopy images were deconvolved using the SoftWorx package (GE Healthcare Life Sciences). Projected images are used for image display. Display of single optical sections is specified in figure legend (Fig 3). We used the SoftWorx measuring tools to determine the fluorescence intensity of line scans. Pixel intensities were plotted along the line as shown in Figs 1D, 1E and 3B. To determine the Tub4-RFP intensity at the SPB (Figs 7D and S4B), we defined a 6 x 6 pixel area as the position of SPB. The mean background intensity was subtracted from the region of interest to yield the net intensity of Tub4-RFP.

### Affinity purification of SPB and mass spectrometry of protein phosphorylation

We used Spc97-TAP and Mps3-TAP for affinity purification of the SPB and Mps3 as reported previously [22, 49]. Briefly, yeast cells were induced to undergo synchronous meiosis for 6 hours; about 10g of cells were harvested and ground in the presence of liquid nitrogen. TAP tagged proteins were then enriched using the epoxy-activated M-270 Dynabeads (Thermo Fisher Scientific, Cat#14305D) that were cross-linked to rabbit IgG (Sigma-Aldrich, Cat#I5006).

Purified SPB proteins were precipitated and digested with trypsin. The resulting peptide mixture was processed through a TiO2-based phosphopeptide enrichment [50]. The resulting enriched phosphorylated peptide samples were directly pressure-loaded onto a C18 column (in-house packed, 15 cm x100 um, 5 μ Gemini C18 resin (Phenomenex)) and were then analyzed by online nanoflow liquid chromatography tandem mass spectrometry (LC-MS/MS) on an Agilent 1200 quaternary HPLC system (Agilent, Palo Alto, CA) connected to an LTQ Orbitrap Velos mass spectrometer (Thermo Fisher Scientific) through an in-house built nanoelectrospray ion source with a linear gradient of 5% to 40% acetonitrile in 0.1% formic acid in 150 min with a flow rate of ~300 nL/min (through split). MS instrument method consisted of one FT full-scan mass analysis (300–1600 m/z, 120,000 resolving power at m/z = 400) followed by 20 data-dependent collision-induced dissociation (CID) MS/MS spectra (normalized collision energy at 35%) with dynamic exclusion for 120 s. Application of mass spectrometer scan functions and HPLC solvent gradients were controlled by the Xcalibur data system (Thermo Fisher Scientific).

Protein identification was carried out with Integrated Proteomics Pipeline—IP2 (Integrated Proteomics Applications, Inc., San Diego, CA. http://www.integratedproteomics.com/). Briefly, MS/MS spectra were extracted using RawXtract (version 1.9.9) [51] and searched with ProLuCID algorithm [52] against a *Saccharomyces cerevisiae* database concatenated to a decoy database in which the sequence for each entry in the original database was reversed [53]. A static modification (+ 57.02146) on cysteine was added into the search due to alkylation of cysteine residues. For phosphopeptide analysis, the ProLuCID search was performed using differential modification of serine and threonine due to phosphorylation (+79.9663). The precursor mass tolerance was set at 30 ppm and fragment mass tolerance was set at 600 ppm. The enzyme specificity was semi-tryptic, with number of missed cleavages at 2. ProLuCID search results were then assembled and filtered using the DTASelect (version 2.0) program [54]. For protein identification, the protein false positive rate was kept below one percent and the average mass deviation was less than 5 ppm. For phosphopeptide identification, only modified peptides were considered and the peptide false positive rate was set at less than one percent.

### Affinity purification of Mps3-TAP and sucrose gradient fractionation

Using a TAP-purification method similar to that described above, we purified Mps3-TAP by affinity purification. To remove the phosphate group from Mps3, purified Mps3-TAP was treated with alkaline phosphatase from calf intestine (CIP, NEB M0290L) at 37°C for 30 min.

Affinity purified Mps3 protein complexes were subject to sucrose gradient fractionation. First, Mps3-TAP was removed from the magnetic beads with TEV protease treatment (0.5mg/ml final concentration) at 16°C for 2 h. Then, cleaved Mps3-TAP* protein samples (note that the protein A peptide domain was now removed from Mps3-TAP) were laid on top of a 7%-47% 12 ml linear sucrose gradient (Gradient Master, Biocomp Instruments) and centrifuged at 39,000 rpm for 18 h at 4°C with the SW41 rotor on a Beckman Ultra 100 centrifuge. Finally, we used an 18G needle to collect 1 ml fractions, the top 9 fractions were shown in Fig 8B. Presence of Mps3 was determined by western blotting.

### Western blotting

Yeast aliquots were withdrawn at indicated times for protein extraction with the trichloroacetic acid (TCA) method as described previously [55]. Briefly, 7 to10 ml of yeast cells were collected, resuspended in 2.5% ice cold TCA, and then incubated at 4°C for 10 minutes. Cell pellets were stored at -80°C, and proteins were extracted by bead beating with a mini bead-beater homogenizer for 1 minute at 4°C. For the mitotic experiments shown in Figs 7 and S4, 2 ml yeast cells were precipitated in the presence of 20 mM NaOH. GFP-tagged proteins were detected by an anti-GFP mouse monoclonal antibody (1:10,000 dilution, Thermo Fisher Scientific, cat#GF28R). HA-tagged proteins were detected by an anti-HA mouse monoclonal antibody (1:10,00 dilution, 12CA5, Sigma). Mps3-V5 was detected by an anti-V5 antibody (1:5,000, Thermo Fisher Scientific, cat#R960-25). We used an anti-TAP antibody (1:10,000, Thermo Fisher Scientific, cat#CAB1001) to determine the presence of Mps3-TAP. This antibody was raised against the C-terminus of the TAP construct after removing the protein A domain by the TEV protease and therefore recognizes TEV-cleaved Mps3-TAP* (Fig 8B). The following synthetic peptide Ac-DKEND(pS)AYEMFKC-amide was used to generate the phosphospecific antibodies against the phosphorylated Mps3-S70 (New England Peptide Inc., Gardner, MA 01440, USA). Raised polyclonal antibodies were affinity purified before use (Fig 4B). A home-made Tub2 antibody [55] was used to detect the level of β-tubulin, and the level of Pgk1 was probed by a Pgk1 antibody (Thermo Fisher Scientific, cat#PA5-28612). The levels of β-tubulin and Pgk1 served as loading controls. Horseradish peroxidase-conjugated secondary antibodies, goat anti-mouse and goat anti-rabbit (Bio-Rad, cat#1706516 and 1705046), were used to probe the proteins of interest by an enhanced chemiluminescence kit (Bio-Rad, cat#1705060).

## Supporting information

S1 Fig The role of proteasome in Mps3 cleavage. (**A**) Western blotting showing N-terminal cleavage product of Mps3. Yeast cells were induced to undergo synchronous meiosis at 33°C, and cell aliquots were withdrawn at indicated time and prepared for western blotting. In these cells, the *GFP-MPS3* construct was under the control of the *DMC1* promoter to express Mps3 more abundantly. Note that Mps3 cleavage is inhibited in *pre9Δ* cells. The level of Pgk1 serves as a loading control. Strains HY4371 and HY5673. (**B**) Cytological evidence of Mps3 cleavage during meiosis. Yeast cells were induced to undergo synchronous meiosis for about 10h, and fluorescence microscopy was performed to determine the intensity of GFP and RFP in yeast tetrads. In these cells, the *GFP-MPS3-RFP* was under the control of its endogenous promoter, and was the only functional copy of *MPS3*. Bright-field images show the morphology of these cells at the end of meiosis. Note that in the absence of Pre9, the GFP signal persists, indicating the lack of N-terminal cleavage. Strains HY5098 and HY5670.
(TIF)

S2 Fig Cytological observation of Mps3 cleavage during yeast meiosis. Yeast cells were induced to undergo synchronous meiosis for 5 hours, and then cells were prepared for live-cell time-lapse microscopy. The expression of *RFP-MPS3-GFP* was under the control of the endogenous *MPS3* promoter. Dashed lines indicate the shape of the cells of interest. Time zero is defined as the start of microscopy. Note that GFP, but not RFP, was retained at the end of meiosis in the cell shown to the right, suggesting the removal of the N-terminal domain from Mps3. The left cell remained at prophase I without separating its SPBs during the entire time course and serves as a control. Strain HY5151.
(TIF)

S3 Fig Phosphorylation of Mps3 at S70 at meiotic prophase I. Yeast cells were induced to undergo synchronous meiosis at 30°C, and cell aliquots were withdrawn at indicated time and prepared for western blotting. The V5 tag was incorporated at the endogenous *MPS3* locus, and *MPS3-V5* served as the only functional copy of *MPS3* in these cells. The level of Pgk1 serves as a loading control. Note that in wild-type cells (also shown in Fig 4C) the level of Mps3-S70 phosphorylation peaked 6h after the induction of meiosis, whereas the cleaved C-terminal fragment of Mps3 peaked 8h after induction. Phosphorylation at S70 occurred in blocked prophase I cells, but cleavage of Mps3 was minimal in these cells. Strains HY4032 and HY5568.
(TIF)

S4 Fig The role of Mps3-S70 phosphorylation during yeast mitosis. (**A**) Representative images showing Mps3-S70D and Mps3-S70A localization in mitosis. Yeast cells were cultured and analyzed as described in **Fig 7**. Like Mps3-nc, Mps3-S70A also accumulated at the nuclear periphery (arrows). Strains HY5372 and HY5373. (**B**) Quantification of fluorescence intensity of Tub4-RFP before SPB separation during mitosis. Error bars represent standard deviation. (**C**) Protein levels of Mps3-S70D and Mps3-S-70A during mitosis. (**D**) Budding index of *mps3-S70D* and *mps3-S70A* mutant cells during mitosis. (**E**) Quantification of SPB separation in *mps3-S70D* and *mps3-S70A* mutant cells during mitosis.
(TIF)

S1 Table Yeast strains used in this study. (DOCX)

S2 Table Plasmids used in this study. (DOCX)

S3 Table PCR primers used in this study. (DOCX)
