# Supplementary figures and images for: Cleavage of the SUN-domain protein Mps3 at its N-terminus regulates centrosome disjunction in budding yeast meiosis
Source: PLoS Genet. 2017 Jun 13;13(6):e1006830. doi: 10.1371/journal.pgen.1006830 (PMC5487077; doi:10.1371/journal.pgen.1006830)

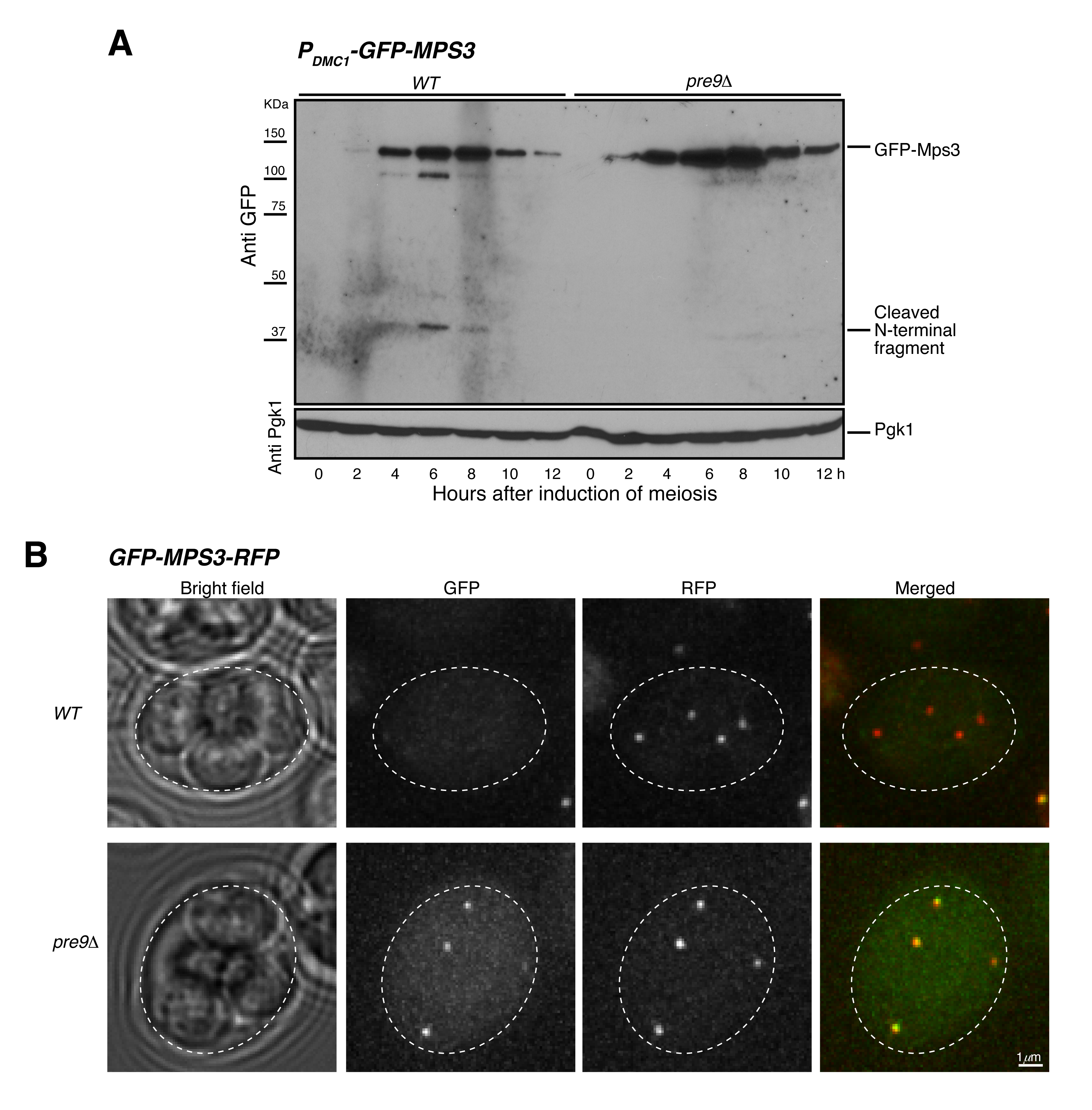

Supplement: S1 Fig — (A) Western blotting showing N-terminal cleavage product of Mps3. Yeast cells were induced to undergo synchronous meiosis at 33°C, and cell aliquots were withdrawn at indicated time and prepared for western blotting. In these cells, the GFP-MPS3 construct was under the control of the DMC1 promoter to express Mps3 more abundantly. Note that Mps3 cleavage is inhibited in pre9Δ cells. The level of Pgk1 serves as a loading control. Strains HY4371 and HY5673. (B) Cytological evidence of Mps3 cleavage during meiosis. Yeast cells were induced to undergo synchronous meiosis for about 10h, and fluorescence microscopy was performed to determine the intensity of GFP and RFP in yeast tetrads. In these cells, the GFP-MPS3-RFP was under the control of its endogenous promoter, and was the only functional copy of MPS3. Bright-field images show the morphology of these cells at the end of meiosis. Note that in the absence of Pre9, the GFP signal persists, indicating the lack of N-terminal cleavage. Strains HY5098 and HY5670. (TIF) [file pgen.1006830.s001.tif]

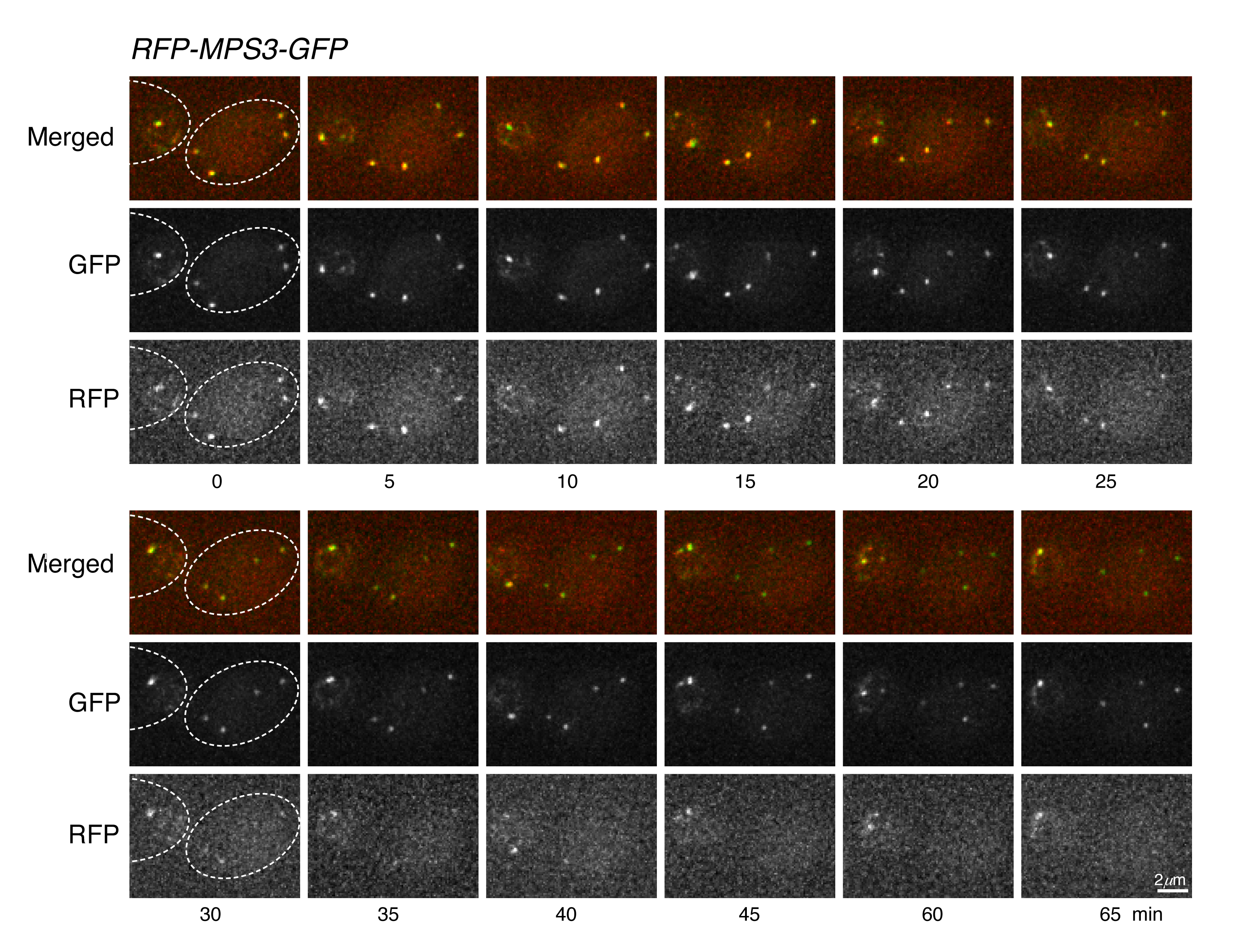

Supplement: S2 Fig — Yeast cells were induced to undergo synchronous meiosis for 5 hours, and then cells were prepared for live-cell time-lapse microscopy. The expression of RFP-MPS3-GFP was under the control of the endogenous MPS3 promoter. Dashed lines indicate the shape of the cells of interest. Time zero is defined as the start of microscopy. Note that GFP, but not RFP, was retained at the end of meiosis in the cell shown to the right, suggesting the removal of the N-terminal domain from Mps3. The left cell remained at prophase I without separating its SPBs during the entire time course and serves as a control. Strain HY5151. (TIF) [file pgen.1006830.s002.tif]

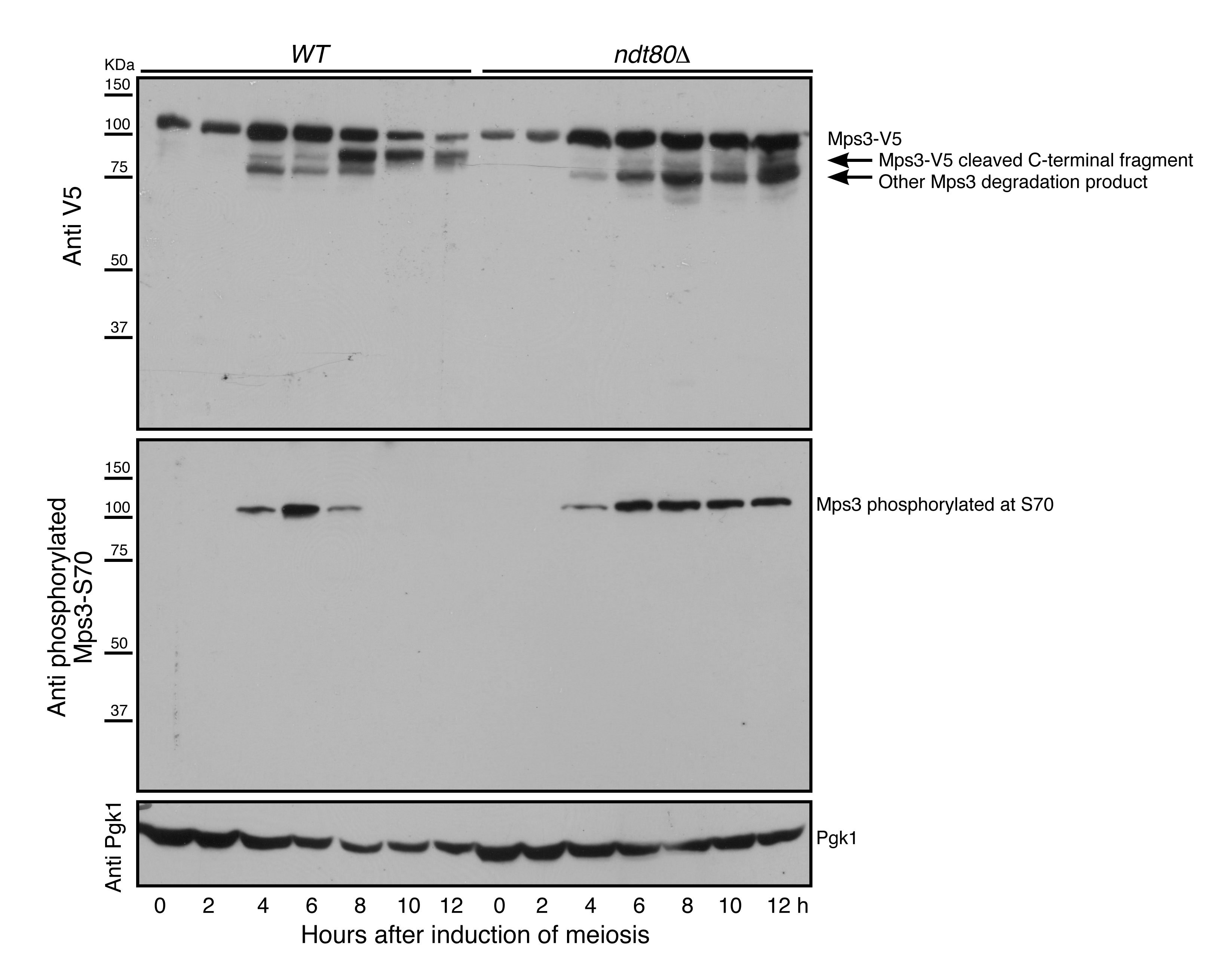

Supplement: S3 Fig — Yeast cells were induced to undergo synchronous meiosis at 30°C, and cell aliquots were withdrawn at indicated time and prepared for western blotting. The V5 tag was incorporated at the endogenous MPS3 locus, and MPS3-V5 served as the only functional copy of MPS3 in these cells. The level of Pgk1 serves as a loading control. Note that in wild-type cells (also shown in Fig 4C) the level of Mps3-S70 phosphorylation peaked 6h after the induction of meiosis, whereas the cleaved C-terminal fragment of Mps3 peaked 8h after induction. Phosphorylation at S70 occurred in blocked prophase I cells, but cleavage of Mps3 was minimal in these cells. Strains HY4032 and HY5568. (TIF) [file pgen.1006830.s003.tif]

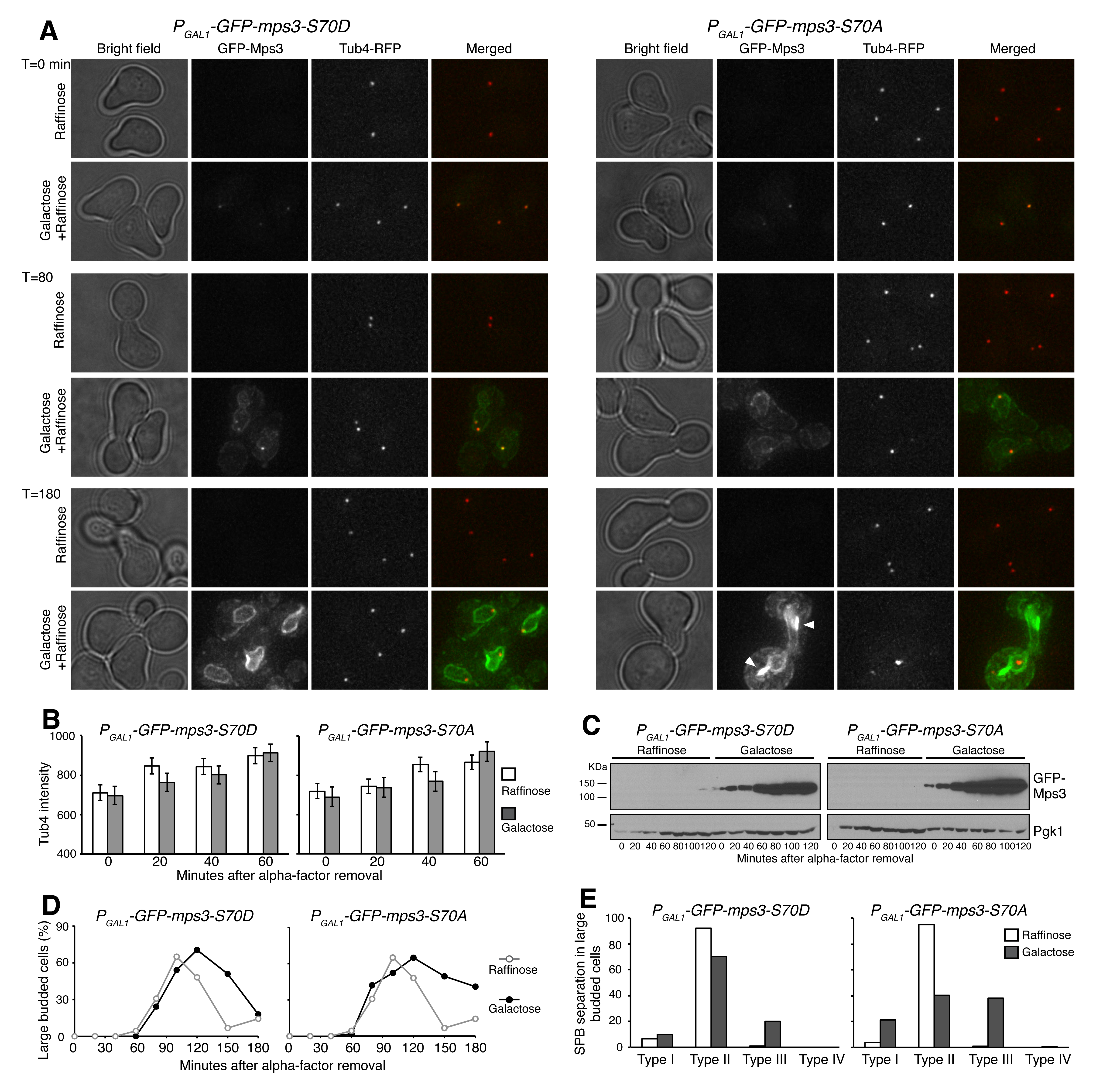

Supplement: S4 Fig — (A) Representative images showing Mps3-S70D and Mps3-S70A localization in mitosis. Yeast cells were cultured and analyzed as described in Fig 7. Like Mps3-nc, Mps3-S70A also accumulated at the nuclear periphery (arrows). Strains HY5372 and HY5373. (B) Quantification of fluorescence intensity of Tub4-RFP before SPB separation during mitosis. Error bars represent standard deviation. (C) Protein levels of Mps3-S70D and Mps3-S-70A during mitosis. (D) Budding index of mps3-S70D and mps3-S70A mutant cells during mitosis. (E) Quantification of SPB separation in mps3-S70D and mps3-S70A mutant cells during mitosis. (TIF) [file pgen.1006830.s004.tif]
