# Supplementary material for: Cleavage of the SUN-domain protein Mps3 at its N-terminus regulates centrosome disjunction in budding yeast meiosis
Source: PLoS Genet. 2017 Jun 13;13(6):e1006830. doi: 10.1371/journal.pgen.1006830 (PMC5487077; doi:10.1371/journal.pgen.1006830)
Supplement: S1 Table — (DOCX) [file pgen.1006830.s005.docx]

Table S1. Yeast strains used in this study

| Name | Background | Genotype |
| --- | --- | --- |
| HY3674 | SK1, diploid | *ura3, leu2, SPC97-TAP::HIS5, SPC72-GFP::HIS5/ura3, leu2, SPC97-TAP::HIS5, SPC72-GFP::HIS5* |
| HY3810 | SK1, diploid | *leu2, ura3, MPS3-TAP::HIS5/leu2, ura3, MPS3-TAP::HIS5* |
| HY3871 | SK1, diploid | *his3∆200, leu2-k, ura3, lys2, ho::LYS2, MPS3-3HA::HIS5/his3∆200, leu2-k, ura3, lys2, ho::LYS2, MPS3-3HA::HIS5* |
| HY4032 | SK1, diploid | *ura3, leu2, MPS3-V5::His5, ndt80∆::KAN, NDJ1-3HA::HIS5/ura3, leu2, MPS3-V5::His5, ndt80∆::KAN, NDJ1-3HA::HIS5* |
| HY4238 | S288C, haploid | *MATa, his3∆1, met15∆0, ura3∆0, MPS3::P_GAL1_-GFP-MPS3::LEU2* |
| HY4346 | S288C, haploid | *MATa, his3∆1, leu2∆0, met15∆0, ura3∆0, TUB4-RFP::HIS5* |
| HY4365 | S288C, haploid | *MATa, his3∆1, leu2∆0, met15∆0, ura3∆0, TUB4-RFP::HIS5, MPS3::P_GAL1_-GFP-mps3-nc::LEU2* |
| HY4371 | SK1, diploid | *leu2, ura3, TUB4-RFP::HIS5, P_DMC1_-GFP-MPS3::LEU2/leu2, ura3, TUB4-RFP::HIS5, P_DMC1_-GFP-MPS3::LEU2* |
| HY4373 | SK1, diploid | *leu2, ura3, TUB4-RFP::HIS5, P_DMC1_-GFP-mps3-nc::LEU2/leu2, ura3, TUB4-RFP::HIS5, P_DMC1_-GFP-mps3-nc::LEU2* |
| HY4394 | SK1, diploid | *his3∆200, leu2-k, ura3, lys2, ho::LYS2, MPS3-3HA::HIS5/leu2, ura3, MPS3-TAP::HIS5* |
| HY4430 | SK1, diploid | *ura3, leu2, his4, P_CLB2_-IPL1::KANMX4, TUB4-RFP::HIS5, ndt80∆::HB, MPS3::P_DMC1_-MPS3::LEU2/ura3, leu2, his4, P_CLB2_-IPL1::KANMX4, TUB4-RFP::HIS5, ndt80∆::HB, MPS3::P_DMC1_-MPS3::LEU2* |
| HY4431 | SK1, diploid | *ura3, leu2, his4, P_CLB2_-IPL1::KANMX4, TUB4-RFP::HIS5, ndt80∆::HB, MPS3::P_DMC1_-MPS3-S70A::LEU2/ura3, leu2, his4, P_CLB2_-IPL1::KANMX4, TUB4-RFP::HIS5, ndt80∆::HB, MPS3::P_DMC1_-MPS3-S70A::LEU2* |
| HY4432 | SK1, diploid | *ura3, his4, P_CLB2_-IPL1::KANMX4, TUB4-RFP::HIS5, ndt80∆::HB, MPS3::P_DMC1_-GFP-mps3-nc::LEU2/ura3,his4, P_CLB2_-IPL1::KANMX4, TUB4-RFP::HIS5, ndt80∆::HB, MPS3::P_DMC1_-GFP-mps3-nc::LEU2* |
| HY4456 | S288C, haploid | *MATa, his3∆1, leu2∆0, met15∆0, ura3∆0, MPS3::P_GAL1_-GFP-mps3-nc::LEU2* |
| HY4468 | SK1, diploid | *ura3, leu2, his4, P_CLB2_-IPL1::KANMX4, TUB4-RFP::HIS5, ndt80∆::HB, MPS3::P_DMC1_-GFP-mps3-S70D::LEU2/ura3, leu2, his4, , P_CLB2_-IPL1::KANMX4, TUB4-RFP::HIS5, ndt80∆::HB, MPS3::P_DMC1_-GFP-mps3-S70D::LEU2* |
| Hy4484 | SK1, diploid | *ura3, leu2, his4, , P_CLB2_-IPL1::KANMX4, TUB4-RFP::HIS5, ndt80∆::HB, MPS3::P_DMC1_-GFP-mps3(∆1-64)::LEU2/ura3, leu2, his4, P_CLB2_-IPL1::KANMX4, TUB4-RFP::HIS5, ndt80∆::HB, MPS3::P_DMC1_-GFP-mps3(∆1-64)::LEU2* |
| HY4824 | SK1, diploid | *ura3, his4, P_CLB2_-IPL1::KANMX4, TUB4-RFP::HIS5, ndt80∆::HB, MPS3::P_DMC1_-GFP-mps3-nc::LEU2, P_DMC1_-TEVprotease::URA3/ura3, his4, P_CLB2_-IPL1::KANMX4, TUB4-RFP::HIS5, ndt80∆::HB, MPS3::P_DMC1_-GFP-mps3-nc::LEU2, P_DMC1_-TEVprotease::URA3* |
| HY4835 | SK1, diploid | *ura3, MPS3::P_DMC1_-GFP-MPS3-RFP::LEU2/ura3, MPS3:: P_DMC1_-GFP-MPS3-RFP::LEU2* |
| HY4917 | S288C, haploid | *MATa, his3∆1, leu2∆0, lys2∆0, ura3∆0, pom152∆::KAN* |
| HY4934 | S288C, haploid | *MATa, his3∆1, leu2∆0, lys2∆0, ura3∆0, pom152∆::KAN, MPS3::P_GAL1_-GFP-MPS3::LEU2* |
| HY4935 | S288C, haploid | *MATa, his3∆1, leu2∆0, lys2∆0, ura3∆0, pom152∆::KAN, MPS3::P_GAL1_-GFP-mps3-nc::LEU2* |
| HY4970 | SK1, diploid | *ura3, leu2, his4, P_CLB2_-IPL1::KANMX4, TUB4-RFP::HIS5, ndt80∆::HB, MPS3::P_DMC1_-GFP-mps3(∆1-93)::LEU2/ura3, leu2, his4, P_CLB2_-IPL1::KANMX4, TUB4-RFP::HIS5, ndt80∆::HB, MPS3::P_DMC1_-GFP-mps3(∆1-93)::LEU2* |
| HY4978 | SK1, diploid | *leu2, ura3, TUB4-RFP::HIS5, MPS3::P_DMC1_-GFP-mps3(∆1-93)::LEU2/leu2, ura3, TUB4-RFP::HIS5, MPS3::P_DMC1_-GFP-mps3(∆1-93)::LEU2* |
| HY5033 | SK1, diploid | *his3∆200, leu2-k, ura3, lys2, ho::LYS2, TUB4-RFP::HIS5, GFP-MPS3/his3∆200, leu2-k, ura3, lys2, ho::LYS2, TUB4-RFP::HIS5, GFP-MPS3* |
| HY5044 | SK1, diploid | *his3∆200, leu2-k, ura3, lys2, ho::LYS2, TUB4-RFP::HIS5, GFP-KAR1/his3∆200, leu2-k, ura3, lys2, ho::LYS2, TUB4-RFP::HIS5, GFP-KAR1* |
| HY5098 | SK1, diploid | *leu2, ura3, GFP-MPS3-RFP::HIS5/leu2, ura3, GFP-MPS3-RFP::HIS5* |
| HY5151 | SK1, diploid | *his3∆200, leu2-k, ura3, RFP-MPS3-GFP/his3∆200, leu2-k, ura3, RFP-MPS3-GFP* |
| HY5268 | SK1, diploid | *ura3, leu2, TUB4-RFP::HIS5, MPS3::P_DMC1_-MPS3::LEU2, P_CLB2_-CDC20::KANMX6/ura3, leu2, TUB4-RFP::HIS5, MPS3::P_DMC1_-MPS3::LEU2, P_CLB2_-CDC20::KANMX6* |
| HY5277 | SK1, diploid | *ura3, leu2, GFP-MPS3::LEU2, NUP49-RFP::HIS5/ura3, leu2, GFP-MPS3::LEU2, NUP49-RFP* |
| HY5372 | S288C, haploid | *MATa, his3∆1, leu2∆0, met15∆0, ura3∆0, TUB4-RFP::HIS5, MPS3::P_GAL1_-GFP-mps3-S70A::LEU2* |
| HY5373 | S288C, haploid | *MATa, his3∆1, leu2∆0, met15∆0, ura3∆0, TUB4-RFP::HIS5, MPS3::P_GAL1_-GFP-mps3-S70D::LEU2* |
| HY5384 | S288C, haploid | *MATa, his3∆1, leu2∆0, met15∆0, ura3∆0, TUB4-RFP::HIS5, MPS3::P_GAL1_-GFP-MPS3::LEU2* |
| HY5464 | SK1, diploid | *leu2-k, ura3, lys2, ho::LYS2, GFP-MPS3, KAR1::P_KAR1_-RFP::LEU2/leu2-k, ura3, lys2, ho::LYS2, GFP-MPS3, KAR1::P_KAR1_-RFP::LEU2* |
| HY5567 | SK1, diploid | *his4-x, ura3, lys2, hoLYS2, leu2hisG, pdr5::KAN/his4-x, ura3, lys2, hoLYS2, leu2hisG, pdr5::KAN, MPS3::P_DMC1_-GFP-MPS3::LEU2* |
| HY5568 | SK1, diploid | *ura3, leu2, his3, MPS3-V5::HIS5//ura3, leu2, his3, MPS3-V5::HIS5* |
| HY5670 | SK1, diploid | *leu2, ura3, GFP-MPS3-RFP::HIS5, pre9∆::KAN//leu2, ura3, GFP-MPS3-RFP::HIS5, pre9∆::KAN* |
| HY5673 | SK1, diploid | *his3∆200, leu2-k, ura3, lys2, ho::LYS2, pre9∆::Kan, P_DMC1_-GFP-MPS3::LEU2//his3∆200, leu2-k, ura3, lys2, ho::LYS2, pre9∆::Kan, P_DMC1_-GFP-MPS3::LEU2* |
| HY5741 | SK1, diploid | *his3∆200, leu2-k, ura3, lys2, ho::LYS2, TUB4-RFP::HIS5, GFP-MPS3/his3∆200, leu2-k, ura3, lys2, ho::LYS2, TUB4-RFP::HIS5, GFP-MPS3* |
| HY5742 | SK1, diploid | *his3∆200, leu2-k, ura3, lys2, ho::LYS2, TUB4-RFP::HIS5, GFP-mps3-nc/his3∆200, leu2-k, ura3, lys2, ho::LYS2, TUB4-RFP::HIS5, GFP-mps3-nc* |
| BY4741 | S288C, haploid | *MATa, his3∆1, leu2∆0, met15∆0, ura3∆0* |
