# Supplementary material for: Cleavage of the SUN-domain protein Mps3 at its N-terminus regulates centrosome disjunction in budding yeast meiosis
Source: PLoS Genet. 2017 Jun 13;13(6):e1006830. doi: 10.1371/journal.pgen.1006830 (PMC5487077; doi:10.1371/journal.pgen.1006830)
Supplement: S2 Table — (DOCX) [file pgen.1006830.s006.docx]

**Table S2.** Plasmids used in this study.

| Plasmid name | Description |
| --- | --- |
| pHG323 | *P_GAL1_-GFP-MPS3, LEU2* |
| pHG348 | *P_GAL1_-GFP-mps3-nc, LEU2* |
| pHG350 | *P_DMC1_-GFP-MPS3, LEU2* |
| pHG358 | *P_GAL1_-GFP-mps3-S70A, LEU2* |
| pHG363 | *P_DMC1_-GFP-mps3-nc, LEU2* |
| pHG375 | *P_DMC1_-GFP-mps3-S70A, LEU2* |
| pHG376 | *P_GAL1_-GFP-mps3-S70D, LEU2* |
| pHG380 | *P_DMC1_-GFP-mps3-S70D, LEU2* |
| pHG431 | *P_DMC1_-TEVprotease, URA3* |
| pHG454 | *P_MPS3_-GFP-MPS3, URA3* |
| pHG459 | *P_MPS3_-GFP-mps3-nc, URA3* |
| pHG465 | *P_KAR1_-GFP-KAR1, URA3* |
| pHG468 | *P_MPS3_-RFP-MPS3, URA3* |
| pHG501 | *P_KAR1_-RFP-KAR1, LEU2* |
