# Supplementary material for: Cleavage of the SUN-domain protein Mps3 at its N-terminus regulates centrosome disjunction in budding yeast meiosis
Source: PLoS Genet. 2017 Jun 13;13(6):e1006830. doi: 10.1371/journal.pgen.1006830 (PMC5487077; doi:10.1371/journal.pgen.1006830)
Supplement: S3 Table — (DOCX) [file pgen.1006830.s007.docx]

**Table S3.** PCR primers used in this study.

| Primer name | Sequence |
| --- | --- |
| MPS3-TAGF | GTTTCATCCCGCTTCTAACGTCCCATCATTTGGCCAAGATGAGCTAGATCAAGCGGCCGCTCTAGAACTAGTGG |
| MPS3-TAGR | GCGATTTTCTGGGGGCCAGGGGGTTAGAACGTTTAATTTTTTATTGTCGTCCCCCTCGAGGTCGACGGTATCG |
| MPS3-COLONYF1 | TGAGACGCGAAAATGGAAT |
| MPS3-COLONYR1 | AGTTGTCAGTTTCACCGCCAT |
| MPS3-COLONYF2 | AAGTGGACGAGAACTACGGGA |
| MPs3-COLONYR2 | AACATCTGAATGCCGTGTGG |
| NUP49-TAGF | GTTACATCAAAAAACGAAAACACTGGCATCATTGAGCATAGCGGCCGCTCTAGAACTAGT |
| NUP49-TAGR | ACTTGTTATACGCACTATATAAACTTTCAGGGCGATTTACCCCCCTCGAGGTCGACGGTA |
| NUP49-COLONYF | CTCCGGGAAGCAAAATTTCT |
| NUP49-COLONYR | TCGTATGTACCTTTTTGCAGG |
| GFP-COLONYF | TGGAGTTGTCCCAATTCTTG |
| PRE9F | ATAACACTCGCAGAGCTCGAT |
| PRE9R | CGCAGACATCGCTTTTGTTT |
